# Reminding Peer Reviewers of Reporting Guideline Items to Improve Completeness in Published Articles: Primary Results of 2 Randomized Trials

**DOI:** 10.1001/jamanetworkopen.2023.17651

**Published:** 2023-06-09

**Authors:** Benjamin Speich, Erika Mann, Christof M. Schönenberger, Katie Mellor, Alexandra N. Griessbach, Paula Dhiman, Pooja Gandhi, Szimonetta Lohner, Arnav Agarwal, Ayodele Odutayo, Iratxe Puebla, Alejandra Clark, An-Wen Chan, Michael M. Schlussel, Philippe Ravaud, David Moher, Matthias Briel, Isabelle Boutron, Sara Schroter, Sally Hopewell

**Affiliations:** 1 Centre for Statistics in Medicine, Nuffield Department of Orthopaedics, Rheumatology and Musculoskeletal Sciences, University of Oxford, Oxford, United Kingdom; 2 CLEAR Methods Center, Division of Clinical Epidemiology, Department of Clinical Research, University Hospital Basel, University of Basel, Basel, Switzerland; 3 PLOS, Public Library of Science, San Francisco, California; 4 The EQUATOR Network, Oxford, United Kingdom; 5 Rehabilitation Sciences Institute, University of Toronto, Toronto, Ontario, Canada; 6 Swallowing Rehabilitation Research Laboratory, Toronto Rehabilitation Institute, University Health Network, Toronto, Ontario, Canada; 7 Cochrane Hungary, Clinical Centre of the University of Pécs, Medical School, University of Pécs, Pécs, Hungary; 8 Department of Public Health Medicine, Medical School, University of Pécs, Pécs, Hungary; 9 Department of Health Research Methods, Evidence, and Impact, McMaster University, Hamilton, Ontario, Canada; 10 Division of General Internal Medicine, Department of Medicine, McMaster University, Hamilton, Ontario, Canada; 11 Division of Nephrology, Toronto General Hospital, University Health Network, Toronto, Ontario, Canada; 12 ASAPbio, Cambridge, United Kingdom; 13 *PLOS ONE*, Public Library of Science, Cambridge, United Kingdom; 14 Department of Medicine, Women’s College Research Institute, Women’s College Hospital, University of Toronto, Toronto, Ontario, Canada; 15 Centre d’Épidémiologie Clinique, Hôpital Hôtel-Dieu, Assistance Publique Hôpitaux de Paris, Paris, France; 16 Université de Paris, CRESS, Inserm, INRA, Paris, France; 17 Centre for Journalology, Clinical Epidemiology Program, Ottawa Hospital Research Institute, Ottawa, Ontario, Canada; 18 School of Epidemiology and Public Health, Faculty of Medicine, University of Ottawa, Ottawa, Ontario, Canada; 19 *The BMJ*, London, United Kingdom; 20 Faculty of Public Health & Policy, London School of Hygiene & Tropical Medicine, London, United Kingdom

## Abstract

**Importance:**

Numerous studies have shown that adherence to reporting guidelines is suboptimal.

**Objective:**

To evaluate whether asking peer reviewers to check if specific reporting guideline items were adequately reported would improve adherence to reporting guidelines in published articles.

**Design, Setting, and Participants:**

Two parallel-group, superiority randomized trials were performed using manuscripts submitted to 7 biomedical journals (5 from the *BMJ* Publishing Group and 2 from the Public Library of Science) as the unit of randomization, with peer reviewers allocated to the intervention or control group.

**Interventions:**

The first trial (CONSORT-PR) focused on manuscripts that presented randomized clinical trial (RCT) results and reported following the Consolidated Standards of Reporting Trials (CONSORT) guideline, and the second trial (SPIRIT-PR) focused on manuscripts that presented RCT protocols and reported following the Standard Protocol Items: Recommendations for Interventional Trials (SPIRIT) guideline. The CONSORT-PR trial included manuscripts that described RCT primary results (submitted July 2019 to July 2021). The SPIRIT-PR trial included manuscripts that contained RCT protocols (submitted June 2020 to May 2021). Manuscripts in both trials were randomized (1:1) to the intervention or control group; the control group received usual journal practice. In the intervention group of both trials, peer reviewers received an email from the journal that asked them to check whether the 10 most important and poorly reported CONSORT (for CONSORT-PR) or SPIRIT (for SPIRIT-PR) items were adequately reported in the manuscript. Peer reviewers and authors were not informed of the purpose of the study, and outcome assessors were blinded.

**Main Outcomes and Measures:**

The difference in the mean proportion of adequately reported 10 CONSORT or SPIRIT items between the intervention and control groups in published articles.

**Results:**

In the CONSORT-PR trial, 510 manuscripts were randomized. Of those, 243 were published (122 in the intervention group and 121 in the control group). A mean proportion of 69.3% (95% CI, 66.0%-72.7%) of the 10 CONSORT items were adequately reported in the intervention group and 66.6% (95% CI, 62.5%-70.7%) in the control group (mean difference, 2.7%; 95% CI, −2.6% to 8.0%). In the SPIRIT-PR trial, of the 244 randomized manuscripts, 178 were published (90 in the intervention group and 88 in the control group). A mean proportion of 46.1% (95% CI, 41.8%-50.4%) of the 10 SPIRIT items were adequately reported in the intervention group and 45.6% (95% CI, 41.7% to 49.4%) in the control group (mean difference, 0.5%; 95% CI, −5.2% to 6.3%).

**Conclusions and Relevance:**

These 2 randomized trials found that it was not useful to implement the tested intervention to increase reporting completeness in published articles. Other interventions should be assessed and considered in the future.

**Trial Registration:**

ClinicalTrials.gov Identifiers: NCT05820971 (CONSORT-PR) and NCT05820984 (SPIRIT-PR)

## Key Points

**Question:**

Does asking peer reviewers to check whether specific reporting guideline items were adequately reported improve the reporting quality in published articles?

**Findings:**

Two randomized trials were conducted in collaboration with publishing journals. Results from 421 randomly assigned manuscripts (intervention: asking peer reviewers to check specific reporting items; control: usual journal practice) did not indicate any improvement in the reporting quality when peer reviewers were asked to check whether specific reporting guideline items were adequately reported.

**Meaning:**

These findings indicate that reminding peer reviewers of specific reporting items is not useful in increasing reporting completeness in published articles.

## Teaser

These 2 randomized trials examined whether asking peer reviewers to check whether specific reporting items were adequately reported in the manuscript they were reviewing had a positive impact on adherence to reporting guidelines in published biomedical journal articles.

## Introduction

Lack of transparent reporting in published articles is a major issue for readers assessing an article to answer a specific question.^1^ For example, if key items, such as the primary outcome, the planned sample size, or the method of allocation concealment, are not adequately described in a randomized clinical trial (RCT), it is difficult for readers to judge the validity and generalizability of the results.^2^ Furthermore, some studies cannot be included in meta-analyses because of inadequate reporting, hindering researchers from generating the best possible evidence.^3^

Reporting guidelines, which have been available since 1994,^4,5^ provide a minimum list of information that must be reported in a published article. These items increase reader comprehension, improve informed clinical decision-making by health professionals, and support the replication of studies or the inclusion of the data in a systematic review.^6^ The EQUATOR (Enhancing the Quality and Transparency of Health Research) Network, consisting of, among others, epidemiologists, methodologists, clinicians, statisticians, and journal editors, actively promotes the development and use of reporting guidelines.^1,5^ These efforts have led to some improvement in the quality of reporting in published articles over time.^7^ However, 2 systematic reviews of reviews that investigated adherence to reporting guidelines found that more than 85% of those studies concluded that reporting is inadequate.^7,8^

Hence, alongside raising awareness of reporting guidelines, interventions with the potential to improve adherence to reporting guidelines need to be assessed. A scoping review^9^ published in 2019 identified 4 RCTs testing interventions that could potentially improve adherence to reporting guidelines. None of the interventions tested looked at whether providing clear and simple instructions to peer reviewers about specific reporting items improved reporting quality. We therefore conducted 2 randomized trials in collaboration with journals in which we tested whether the low-cost intervention of asking peer reviewers to check whether specific reporting items were adequately reported in the manuscript they were reviewing had a positive impact on the adherence to reporting guidelines in published biomedical journal articles.

## Methods

### Design

Detailed methods of both trials are described in the publicly available study protocols (Supplement 1 and Supplement 2).^10,11^ Briefly, we conducted 2 superiority, parallel-group (2 arms; 1:1 randomization) randomized trials in collaboration with biomedical journals, using submitted manuscripts as the unit of randomization with reviewers allocated to the intervention or control group. The first trial included manuscripts under review that presented RCT results, and the intervention consisted of reminding peer reviewers of the 10 most important and poorly reported CONSORT (Consolidated Standards of Reporting Trials)^12,13^ items by email (the CONSORT for Peer Review [CONSORT-PR] trial). The second trial included manuscripts under review that presented RCT protocols, and the intervention consisted of reminding peer reviewers of the 10 most important and poorly reported Standard Protocol Items: Recommendations for Interventional Trials (SPIRIT)^14,15^ items (the SPIRIT for Peer Review [SPIRIT-PR] trial). The CONSORT-PR trial included manuscripts that described RCT primary results. The SPIRIT-PR trial included manuscripts that contained RCT protocols. Both trials received ethical approval from the Medical Sciences Interdivisional Research Ethics Committee of the University of Oxford and were prospectively registered on Open Science Framework.^16,17^ Of note, registration of the 2 trials in a clinical trial registry was denied by trial registries, such as ClinicalTrials.gov or ISRCTN, because our studies did not measure a health outcome in individuals. After a request by the editors who provided personal contact information to ClinicalTrials.gov representatives, both studies were retrospectively registered on ClinicalTrials.gov (NCT05820971 [CONSORT-PR] and NCT05820984 [SPIRIT-PR]). We report both trials adhering to the CONSORT reporting guideline.^12^

### Eligibility Criteria and Recruitment

For the CONSORT-PR trial, eligible manuscripts needed to report primary results of an RCT. For the SPIRIT-PR trial, eligible manuscripts needed to report an RCT protocol (detailed eligibility criteria for both trials are presented in eAppendix 1 in Supplement 3). Participating journals (CONSORT-PR: *BMJ Open*, *The BMJ*, *British Journal of Sports Medicine*, *British Journal of Ophthalmology*, *Heart*, *PLOS Medicine*, and *PLOS ONE*; SPIRIT-PR: *BMJ Open*) provided automated reports of new submissions to allow daily screening to flag eligible manuscripts. Manuscripts that were flagged as eligible were randomized if they were sent for peer review, with randomization occurring after the first peer reviewer accepted the invitation.

### Interventions

In both trials, control group manuscripts received the usual peer review practice used by the journal they were submitted to. For manuscripts in the intervention group of the CONSORT-PR trial, peer reviewers were sent an additional email (alongside usual journal practice) from the editorial office. The email listed the 10 most important and poorly reported CONSORT items together with a brief description of each and asked reviewers to check whether these items were addressed in the manuscript and to ask authors to include items that were not adequately reported (see email example in eAppendix 2 in Supplement 3). For the SPIRIT-PR trial, the intervention was identical, but instead of CONSORT items, it included the 10 most important and poorly reported SPIRIT items (list of selected items in eTable 1 in Supplement 3). The selection of the 10 most important and poorly reported items for CONSORT-PR was completely based on previous literature.^18^ For SPIRIT-PR, we considered all available assessments of reporting^19,20,21^ and chose 10 items through a consensus process within the study team. More details on this process as well as the development of a brief description for each item is presented in eAppendix 3 in Supplement 3. For manuscripts in the intervention group, we checked daily whether new peer reviewers had accepted the invitation to review and then sent them the additional email through the manuscript tracking systems of the participating journal. Specifically labeled peer reviewers representing patients or the public were excluded from the intervention because they received a different set of questions from the journal.

### Randomization and Blinding

Eligible manuscripts were randomized as soon as the first peer reviewer accepted the invitation to peer review the article. Randomization was conducted by the corresponding author (B.S.) through the Study-Randomizer system^22^ using a 1:1 allocation (random block sizes between 2 and 8) and stratification by journal (for CONSORT-PR). Authors and peer reviewers were not informed about the study. Editors were not actively informed about the randomization. Outcomes assessors were blinded and independently assessed, in duplicate, the adequacy of reporting in the published version of the articles. Any disagreements between outcome assessors were resolved by discussion.

### Outcomes

The primary outcome was the difference between the intervention and control groups in the mean proportion of the 10 selected reporting items that were adequately reported in the final published articles. Secondary outcomes were (1) the mean proportion difference of each adequately reported item considering each of the 10 selected reporting items separately; (2) the mean proportion difference of each adequately reported intervention item, considering their respective subitems (ie, some items consisted of several subitems, see eAppendix 2 and eTable 1 in Supplement 3) as a separate item; (3) the time from assigning an editor to the first decision communicated to authors; (4) the proportion of manuscripts rejected after the first round of peer review; and (5) the proportion of manuscripts published and included for analyses.

### Statistical Analysis

Based on a 2-tailed *t* test, we calculated that 166 (83 per group) published articles were required for CONSORT-PR (type 1 error rate of 5% and a power of 80%) and 106 (53 per arm) for SPIRIT-PR (type 1 error rate of 5% and a power of 90%; see detailed underlying rationale and assumptions in eAppendix 4 in Supplement 3). Because the sample size was driven by the number of articles published rather than the number of manuscripts randomized, we recruited eligible manuscripts until we reached the anticipated number of published articles in each group.

The primary outcome, assessing for a difference in the mean proportion of adequately reported items, was analyzed using an unpaired *t* test. Prespecified subgroup analyses assessed the effect stratified by planned sample size (≥100 vs <100) and journal impact factor (≥10 vs <10; only for CONSORT-PR). Items that consisted of several subitems were considered as adequately reported if all applicable subitems were reported. A post hoc sensitivity analysis was conducted excluding all manuscripts from the randomization that were erroneously included and for which all peer reviewers submitted their reports before the intervention email could be sent. The outcome of time from assigning an editor to the first decision communicated to authors was compared using the Wilcoxon-Mann-Whitney test because visual inspection of data distribution indicated a nonnormal distribution (eFigure in Supplement 3). The analyses for outcomes that assessed reporting completeness considered the published manuscripts as the population for analysis. Therefore, we excluded randomized manuscripts that were not published. For the end points that assessed the proportion of accepted articles and the proportion of articles rejected after the first round of review, all randomized manuscripts were included. Only the end point of time from assigning an editor to the first decision communicated to authors could be analyzed considering all randomized manuscripts as well as only the ones that were published (as prespecified within the study protocol^10^).

## Results

**Figure 1.**
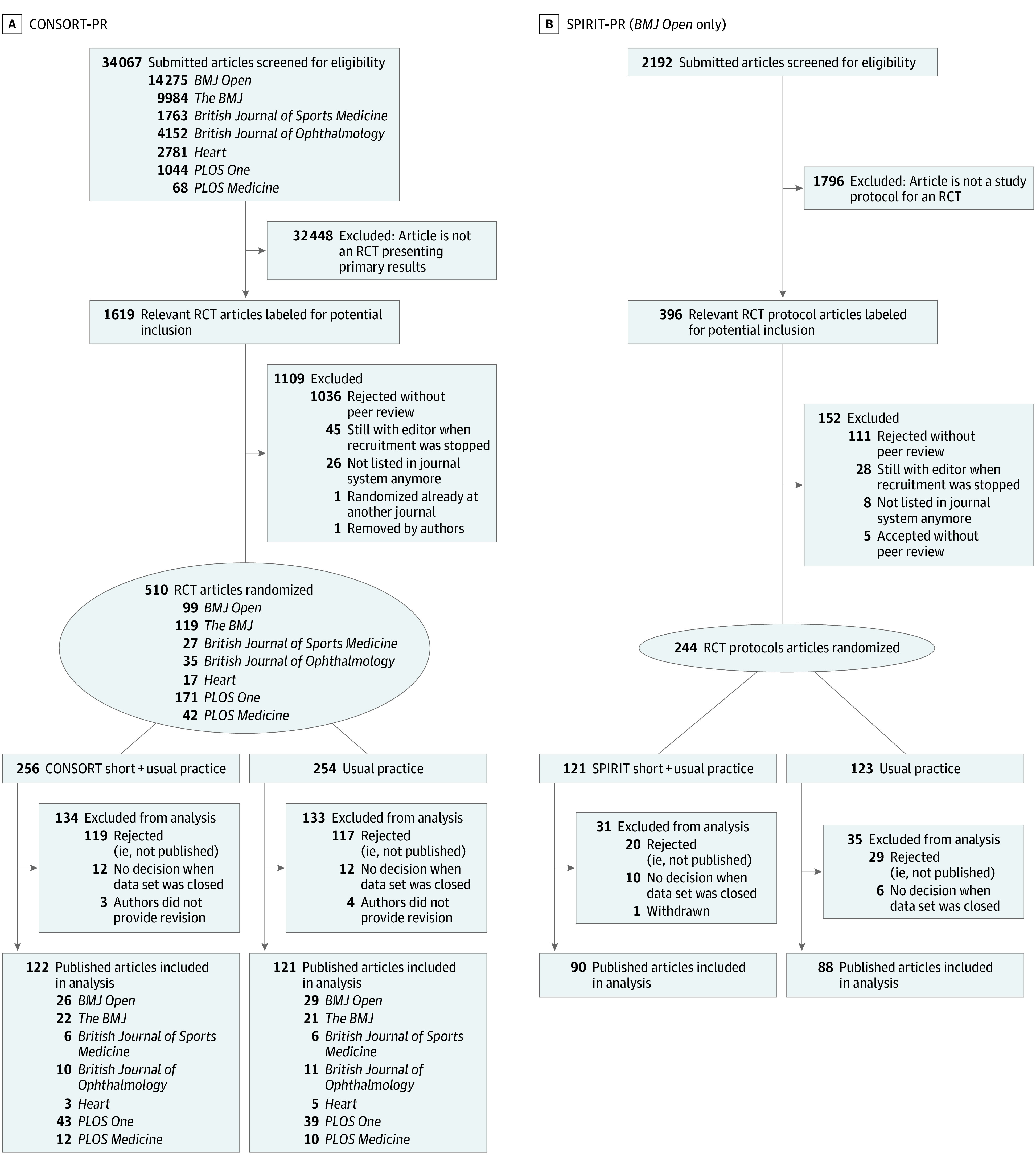
Flowcharts for the CONSORT for Peer Review (CONSORT-PR) and SPIRIT for Peer Review (SPIRIT-PR) Trials RCT indicates randomized clinical trial.

For CONSORT-PR, a total of 34 067 manuscripts were assessed for eligibility by the corresponding author (B.S.) between July 2019 and July 2021 based on title, abstracts, and full texts if necessary. Of those, 510 eligible manuscripts were randomized, and 243 were published and included in the analysis (Figure 1A; eTable 2 in Supplement 3 for detailed stratification by journal). For SPIRIT-PR, 2193 manuscripts were screened, 245 randomized, and 178 included in the analysis (Figure 1B).

The median (IQR) planned sample size of the described trials included was 214 (80-600) in the CONSORT-PR trial and 218 (110-640) in the SPIRIT-PR trial (Table 1). Most included manuscripts presented studies with a superiority (CONSORT-PR: 224 of 243 [92.2%]; SPIRIT-PR: 154 of 178 [86.5%]), parallel-group design (CONSORT-PR: 189 of 243 [77.8%]; SPIRIT-PR: 147 of 178 [82.6%]) using 2 trial groups (CONSORT-PR: 187 of 243 [77.0%]; SPIRIT-PR: 141 of 178 [84.8%]). Few industry-sponsored trials were included (CONSORT-PR: 12 of 243 [4.9%]; SPIRIT-PR: 11 of 178 [6.2%]), and the most commonly assessed interventions were behavioral (CONSORT-PR: 80 of 243 [32.9%]; SPIRIT-PR: 61 of 178 [34.3%]) or drug (CONSORT-PR: 49 of 243 [20.2%]; SPIRIT-PR: 52 of 178 [29.2%]) interventions (Table 1). Medical specialties are listed in eTable 3 in Supplement 3. In general, baseline characteristics were equally distributed between the intervention and control groups.

**Table 1.** Baseline Characteristics of Manuscripts Included in the Study^a^

Characteristic	CONSORT-PR			SPIRIT-PR		
	Intervention group (reminder sent to peer reviewers) (n = 122)	Control group (n = 121)	Total (N = 243)	Intervention group (reminder sent to peer reviewers) (n = 90)	Control group (n = 88)	Total (N = 178)
Planned sample size, median (IQR)	250 (72-705)	200 (90-510)	214 (80-600)	210 (106-528)	220 (110-700)	218 (110-640)^b^
Hypothesis						
Superiority	114 (93.4)	110 (90.9)	224 (92.2)	78 (87.7)	75 (85.2)	154 (86.5)
Noninferiority or equivalence	5 (4.1)	6 (5.0)	11 (4.5)	9 (10.0)	8 (9.1)	17 (9.6)
Superiority and noninferiority	1 (0.8)	0	1 (0.4)	2 (2.2)	4 (4.6)	6 (3.4)
Unclear or labeled differently	2 (1.6)	5 (4.1)	7 (2.9)	0	1 (1.1)	1 (0.6)
Design						
Parallel group	92 (75.4)	97 (80.2)	189 (77.8)	73 (81.1)	74 (84.1)	147 (82.6)
Cluster	20 (16.4)	16 (13.2)	36 (14.8)	9 (10.0)	7 (8.0)	16 (9.0)
Crossover	3 (2.5)	4 (3.3)	7 (2.9)	2 (2.2)	3 (3.4)	5 (2.8)
Factorial	2 (1.6)	0 (0)	2 (0.8)	2 (2.2)	4 (4.6)	6 (3.4)
Other	5 (4.1)	4 (3.3)	9 (3.7)^c^	4 (4.4)	0 (0)	4 (2.2)^d^
Centers						
Single center	53 (43.4)	51 (42.2)	104 (42.8)	29 (32.2)	23 (26.1)	52 (29.2)
Multicenter	67 (54.9)	67 (55.4)	134 (55.1)	61 (67.8)	65 (73.9)	126 (70.8)
Unclear	2 (1.6)	3 (2.5)	5 (2.1)	0	0	0
Trial arms						
2	97 (79.5)	90 (74.4)	187 (77.0)	76 (84.4)	75 (85.2)	151 (84.8)
3	17 (13.9)	22 (18.2)	39 (16.1)	10 (11.1)	8 (9.1)	10 (10.1)
4	7 (5.7)	8 (6.6)	15 (6.2)	4 (4.4)	5 (5.7)	9 (5.1)
Other	1 (0.8)	1 (0.8)	2 (0.8)^e^	0	0	0
Sponsor						
Nonindustry	120 (98.4)	111 (91.7)	231 (95.1)	83 (92.2)	84 (95.5)	167 (93.8)
Industry	2 (1.6)	10 (8.3)	12 (4.9)	7 (7.8)	4 (4.5)	11 (6.2)
Intervention						
Behavioral, lifestyle, education, or counselling	38 (31.2)	42 (34.7)	80 (32.9)	32 (35.6)	29 (33.0)	61 (34.3)
Drug	21 (17.2)	29 (24.0)	50 (20.6)	25 (27.8)	27 (30.7)	52 (29.2)
Device	19 (15.6)	14 (11.6)	33 (13.6)	10 (11.1)	8 (9.1)	18 (10.1)
Other^f^	44 (36.1)	36 (29.8)	80 (32.9)	23 (25.6)	24 (27.3)	47 (26.4)

Abbreviations: CONSORT-PR, CONSORT for Peer Review; SPIRIT-PR, SPIRIT for Peer Review.

^a^ Data are presented as number (percentage) of manuscripts unless otherwise indicated.

^b^ n = 174.

^c^ Two split body, 4 stepped wedge, 1 several subsequent randomizations, 1 single-arm trial, and 1 factorial cluster trial.

^d^ Two stepped wedge designs, 1 split body, and 1 with 2 subsequent randomizations.

^e^ One erroneously included single-arm trial and 1 trial with multiple subsequent randomizations (ie, >4 arms).

^f^ Others are as follows: CONSORT-PR: different approaches, procedures, orders, or process optimization (n = 16); additional diagnostic tests (n = 13); surgery or surgery or drug (n = 10); herbal (n = 6); psychological (n = 6); radiation (n = 6); dietary supplements (n = 5); biological or vaccine (n = 5); acupuncture (n = 4); physical therapy (n = 3); nutrition or hydration (n = 2); genetic (n = 1); different language (n = 1); different exercise (n = 1); interdisciplinary approach (n = 1); SPIRIT-PR: surgery (n = 12); different approaches, procedures, orders, or process optimization (n = 6); psychological (n = 5); biological or vaccine (n = 4); dietary supplements (n = 3); radiation (n = 3); additional diagnostic tests (n = 3); wound management (n = 3); acupuncture (n = 2); stimulation (magnetic or electric; n = 2); physical therapy (n = 1); diet (n = 1); drugs and education (n = 1); infrastructure (n = 1).

In the CONSORT-PR trial, the mean proportion of adequate reporting of the 10 selected CONSORT items (primary outcome) was 69.3% (95% CI, 66.0%-72.7%) in the intervention group and 66.6% (95% CI, 62.5%-70.7%) in the control group (mean difference, 2.7%; 95% CI, −2.6% to 8.0%) (Table 2). We conducted a sensitivity analysis that excluded manuscripts for which all peer reviewers returned their reports before the intervention email could be sent (n = 1) and manuscripts that were erroneously included because it was unclear based on the abstract that they did not present the primary results of an RCT (n = 4; 2 no primary results, 1 study protocol instead of results, and 1 no randomized trial); this analysis revealed comparable results (eTable 4 in Supplement 3). Consideration of each subitem (n = 19) as a separate item also resulted in similar proportions of adequate reporting for the intervention group (78.1%; 95% CI, 75.2%-81.0%) and the control group (76.0%; 95% CI, 72.6%-79.3%; mean difference, 2.3%; 95% CI, −2.3% to 6.5%) (Table 2).

**Table 2.** Difference in the Mean Proportion of Adequate Reporting After Allocating Half of the Manuscripts to an Intervention in Which Peer Reviewers Were Reminded of Selected Reporting Items

Outcome	Intervention group (reminder sent to peer reviewers), % (95% CI) (122 in CONSORT-PR and 90 in SPIRIT-PR)	Control group, % (95% CI) (121 in CONSORT-PR and 88 in SPIRIT-PR)	Mean difference, % (95% CI)	*P* value
**CONSORT-PR trial**				
Proportion of 10 adequately reported CONSORT items (primary outcome)	69.3 (66.0 to 72.7)	66.6 (62.5 to 70.7)	2.7 (−2.6 to 8.0)	.31
Proportion of 10 adequately reported CONSORT items, considering each subitem (n = 19) as a separate item	78.1 (75.2 to 81.0)	76.0 (72.6 to 79.3)	2.2 (−6.5 to 2.3)	.34
**SPIRIT-PR trial**				
Proportion of 10 adequately reported SPIRIT items (primary outcome)	46.1 (41.8-50.4)	45.6 (41.7 to 49.4)	0.5 (−5.2 to 6.3)	.85
Proportion of 10 adequately reported SPIRIT items, considering each subitem (n = 23) as a separate item	69.8 (67.2 to 72.4)	68.7 (65.8 to 71.7)	1.1 (−2.8 to 5.0)	.59

Abbreviations: CONSORT-PR, CONSORT for Peer Review; SPIRIT-PR, SPIRIT for Peer Review.

**Figure 2.**
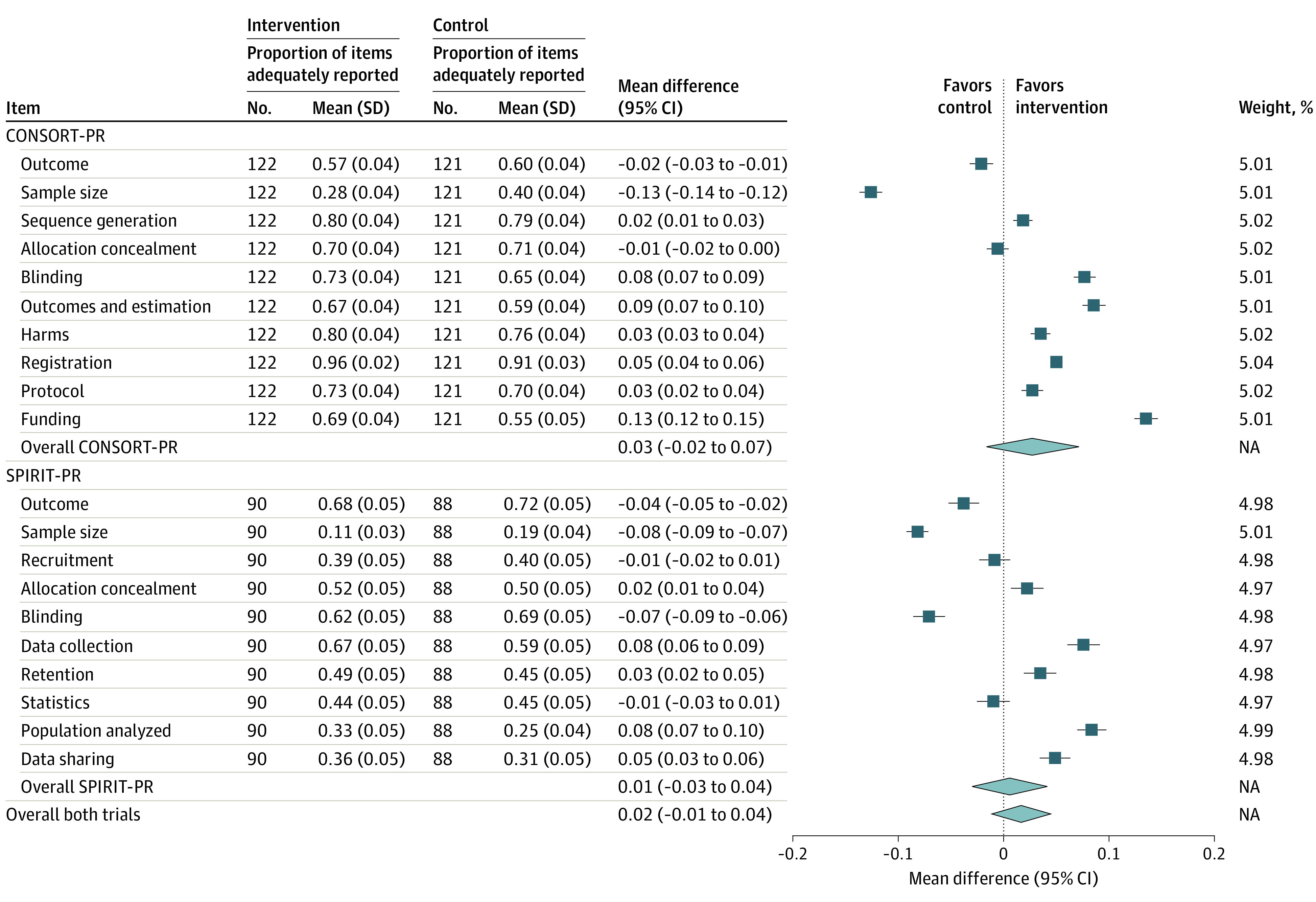
Difference in the Mean Proportion of Adequately Reported CONSORT (Consolidated Standards of Reporting Trials) and SPIRIT (Standard Protocol Items: Recommendations for Interventional Trials) Items in the CONSORT for Peer Review (CONSORT-PR) and SPIRIT for Peer Review (SPIRIT-PR) Trials NA indicates not applicable.

Likewise, the SPIRIT-PR trial showed that sending the additional email did not increase the proportion of adequately reported items (intervention group: 46.1%; 95% CI, 41.8%-50.4%; control group: 45.6%; 95% CI, 41.7%-49.4%; mean difference, 0.5%; 95% CI, −5.2% to 6.3%). Considering each subitem (n = 23) of the 10 selected SPIRIT items separately did not change the results (Table 2). In the SPIRIT-PR trial, no manuscripts were erroneously included, and for each manuscript in the intervention group, at least 1 reviewer received the additional email before submitting their peer review report. Hence, no sensitivity analysis was conducted. The proportion of adequate reporting for each individual reporting item stratified by treatment group is presented in Figure 2. Subgroup analyses of the primary end point indicated better reporting for articles with larger planned sample sizes and trials published in journals with higher impact factors, but none of these subgroup analyses showed a clear benefit when peer reviewers were sent an email reminding them of the most important reporting items (eTable 5 in Supplement 3).

In the CONSORT-PR trial, 122 of 256 articles (47.7%) in the intervention group were accepted and could be included in the analyses vs 121 of 254 (47.6%) in the control group (mean difference, 0.0%; 95% CI, −8.7% to 8.7%) (Table 3). The proportion of accepted articles was higher in the SPIRIT-PR trial, with no clear difference between the intervention group (90 of 121 [74.4%]) and control group (88 of 123 [71.5%]; mean difference, 2.8%; 95% CI, −8.3% to 14.0%). In the CONSORT-PR trial, 109 of 256 articles (42.6%) in the intervention group and 103 of 254 articles (40.6%) in the control group were rejected after the first round of peer review. In the SPIRIT-PR trial, 15 of 121 articles (12.4%) in the intervention group and 25 of 123 articles (20.3%) in the control group were rejected after the first round of peer review. In both trials, no clear difference between the intervention and control groups was seen for the median time from assigning an editor to communication of the first decision (Table 3). Data sets and explanations of the variables for all the trials are given in eTables 6 through 9 in Supplement 4 (code for the primary end point is in eAppendix 5 in Supplement 3).

**Table 3.** Acceptance Rates and Interval Between Editor Assigned Until First Decision Is Reached for Manuscripts Included in the CONSORT-PR and SPIRIT-PR Trials

	Intervention group (reminder to peer reviewers)	Control group	Mean difference (95% CI)	P value
**CONSORT-PR trial**				
Articles that were published and included in analysis, No./total No. (%)	122/256 (47.7)	121/254 (47.6)	0.0 (−8.7 to 8.7)	>.99
Articles rejected after the first round of peer review, No./total No. (%)	109/256 (42.6)	103/254 (40.6)	2.0 (−6.5 to 10.6)	.64
Interval between editor assigned and first decision, median (IQR), d	51 (33 to 74) (n = 256)	54 (30 to 94) (n = 254)	NA	.43
Interval between editor assigned and first decision, considering only articles that were included in the analyses, median (IQR), d	51 (33 to 74; n = 122)	59 (29 to 106) (n = 121)	NA	.23
**SPIRIT-PR trial**				
Articles that were published and included in analysis, No./total No. (%)	90/121 (74.4)	88/123 (71.5)	2.8 (−8.3 to 14.0%)	.62
Articles rejected after the first round of peer review, No./total No. (%)	15/121 (12.4)	25/123 (20.3)	7.9 (−1.3 to 17.2)	.09
Interval between editor assigned and first decision, median (IQR), d	116 (86 to 150) (n = 121)	112 (86 to 149) (n = 123)	NA	.98
Interval between editor assigned and first decision, considering only articles that were included in the analyses, median (IQR), d	116 (83 to 152) (n = 90)	109 (81 to 142) (n = 88)	NA	.70

Abbreviations: CONSORT-PR, CONSORT for Peer Review; NA, not applicable; SPIRIT-PR, SPIRIT for Peer Review.

## Discussion

We have generated strong evidence that asking peer reviewers to check whether reporting items were adequately reported in manuscripts did not substantially improve the completeness of reporting in published articles. This result is in line with a recently published stepped-wedged RCT conducted by Jones et al^23^ that assessed a specific intervention targeted at peer reviewers. They found that providing the primary outcome definition from clinical trial registries to peer reviewers did not increase the agreement of the outcome definition between registry and publication.^23^ On the basis of the results from our 2 randomized trials and from the RCT by Jones et al,^23^ which shows that targeting peer reviewers does not improve reporting, we can speculate about why these interventions did not have an impact. First, it seems likely that peer reviewers already have a high workload (for which they do not receive compensation) and might therefore not be willing to conduct more tasks or follow further instructions. Second, peer reviewers usually are experts who have published in a similar field as the authors^24^ and are therefore not necessarily more experienced in using reporting guidelines than the authors themselves. On the other hand, it is possible that peer reviewers in the intervention group commented more on the reporting items listed than the control group, but the comments were not addressed appropriately by the authors. To answer these questions, we have collected peer reviewer comments from a subsample, which we will analyze in a separate in-depth qualitative study.

A similar RCT published in 2016 by Hopewell et al^18^ assessed the effect of providing a writing tool to authors and also found no effect in improving the completeness of reporting in published articles. The intervention that did show a strong improvement in completeness of reporting (tested within 2 RCTs) was when an additional expert reviewer persistently checked adherence to reporting guidelines.^25,26^ This intervention, however, requires that journals invest in hiring expert reviewers to check adherence to reporting guidelines. The journal *Trials* has implemented such expert reviewers,^27^ and other journals should follow this lead to increase the reporting quality of articles in their journals.

### Strengths and Limitations

Several studies have been conducted to assess whether specific interventions can improve reporting completeness in published articles, however, most of these studies did not use a randomized design.^9^ Our intervention was purposely designed so that it could easily be implemented by journals at low cost if shown to be effective. Conducting 2 trials in parallel allowed us to generate high-quality evidence to answer the question of whether this low-cost intervention of reminding peer reviewers of the most important reporting items could improve reporting completeness in published articles. The following limitations are worth mentioning. First, we do not know what input the intervention had on manuscripts that were not published. In theory, it would have been possible that the intervention had an impact on the acceptance rate, which would have distorted the balance of baseline characteristics between the intervention and control groups. However, given that we did not find a difference in acceptance and rejection rates between the groups and that the baseline characteristics of included manuscripts were well balanced, we are reassured that we can trust the findings of our primary outcome. Furthermore, from the perspective of the readers of journal articles and the publishing journal, one could also argue that only the reporting quality of manuscripts that are actually published is relevant. Nevertheless, to get a better understanding of the impact of the intervention on manuscripts that were not published, we plan an in-depth qualitative study to assess peer reviewer comments from a subsample of randomized manuscripts (both accepted and rejected) to investigate whether manuscripts having more comments about inadequate reporting were more frequently rejected. Second, it is possible that we had a ceiling effect with little room for improvement in reporting quality. This might have occurred in CONSORT-PR, for which we found moderate to good reporting (nearly 70%), but not for SPIRIT-PR, for which the reporting was below 50%. Furthermore, we did not observe higher mean proportions of adequate reporting than we expected in our sample size calculations (eAppendix 4 in Supplement 3). Third, because of technical restrictions, we could not implement our reminder to peer reviewers within the general instructions that they receive from the journal when accepting the review invitation. It is possible that the peer reviewers did not notice the additional email or received the email too late. However, because of our daily screening activities, we were able to send the intervention email in a timely manner, and there was only 1 manuscript in the intervention group for which the peer reviewers did not receive the email in time (eTable 4 in Supplement 3). Fourth, although we collaborated for these 2 trials with 7 journals (convenience sample of journals in general medicine as well as specialist journals), we cannot be certain that the results would be the same in other journals (eg, journals with a higher proportion of industry-sponsored trials). Nonetheless, we believe that the peer review process is comparable in most biomedical journals and that the pool of peer reviewers is not completely different, so we would expect similar findings. In addition, when we consider our subgroup analyses stratified by impact factor, even though we found differences in the overall reporting, the effect of the intervention remained the same (ie, the intervention did not have an effect).

## Conclusions

These 2 randomized trials found that giving peer reviewers an additional task by emailing them a reminder of the 10 most important and poorly reported reporting items did not improve the reporting completeness in published articles. We therefore encourage journals to implement other interventions that have proven to be efficient in other trials (ie, hiring expert reviewers for adherence to reporting guidelines) to increase the reporting completeness in published articles.

## Notes

Supplement 1.
CONSORT-PR Study Protocol

Supplement 2.
SPIRIT-PR Study Protocol

Supplement 3. **eAppendix 1.** Eligibility Criteria for CONSORT-PR and SPIRIT-PR **eAppendix 2.** Example of the Intervention Email Sent in the CONSORT-PR Study for BMJ Open **eAppendix 3.** Selection of the 10 Most Important and Poorly Reported Reporting Items and Development of the Brief Explanation for Each Item **eAppendix 4.** Assumptions for Sample Size Calculations **eAppendix 5.** Stata Code for the Primary Outcome **eTable 1.** The Ten Most Important and Underreported SPIRIT Items as Defined by a Group of Experts **eTable 2.** Proportion of Manuscripts Included in the Analysis of the CONSORT-PR Trial Per Participating Journal **eTable 3.** Medical Specialties of Included Manuscripts **eTable 4.** Sensitivity Analysis of the Primary Outcome for the CONSORT-PR Trial **eTable 5.** The Difference in the Mean Proportion of Adequate Reporting of the 10 Selected Reporting Items, Stratified by Sample Size and Journal Impact Factor **eFigure.** Histograms
eReferences

Supplement 4. **eTable 6.** Data Set From the CONSORT-PR Study, Excluding Variables That Would Identify Individual Studies or Journals **eTable 7.** Explanation of Variables Used in CONSORT-PR **eTable 8.** Data Set from the SPIRIT-PR Study, Excluding Variables That Would Identify Individual Studies **eTable 9.** Explanation of Variables Used in SPIRIT-PR

Supplement 5.
Data Sharing Statement
