# Supplementary material for: Reminding Peer Reviewers of Reporting Guideline Items to Improve Completeness in Published Articles: Primary Results of 2 Randomized Trials
Source: JAMA Netw Open. 2023 Jun 9;6(6):e2317651. doi: 10.1001/jamanetworkopen.2023.17651 (PMC10257091; doi:10.1001/jamanetworkopen.2023.17651)
Supplement: Supplement 1. — CONSORT-PR Study Protocol [file jamanetwopen-e2317651-s001.pdf]

Impact of a short form of the CONSORT checklist for peer reviewers to improve the reporting of randomised controlled trials published in biomedical journals: a randomised controlled trial

Short title: CONSORT for Peer Review (CONSORT-PR)

Principal investigator: Dr Benjamin Speich
Centre for Statistics in Medicine, University of Oxford

Dr Benjamin Speich
Centre for Statistics in Medicine,
Botnar Research Centre, University of Oxford, Windmill Road, Oxford OX3 7LD
Email: Benjamin.speich@ndorms.ox.ac.uk

SCIENTIFIC COMMITTEE

Prof Isabelle Boutron, Paris Descartes University, France
Prof Matthias Briel, University of Basel, Switzerland
Associate Prof Sally Hopewell, Centre for Statistics in Medicine, Oxford University, UK
Prof David Moher, Centre for Journalology, Clinical Epidemiology Program, Ottawa Hospital Research Institute, Canada
Prof Philippe Ravaud, Paris Descartes University, France
Dr Benjamin Speich, Centre for Statistics in Medicine, University of Oxford, UK
Dr. Michael M Schlussel, Centre for Statistics in Medicine, University of Oxford, UK
Dr Sara Schroter, The BMJ, London, UK

Trial registration: This trial will be prospectively registered under clinicaltrials.gov.

Protocol version: Version 1.1 2019-05-21

Funding: Benjamin Speich is supported by an Advanced Postdoc.Mobility grant from the Swiss National Science Foundation (P300PB_177933). David Moher is supported by a University Research Chair, Ottawa. The funders had no role in designing the study and will also have no role in conducting the study as well as in analysing and reporting study results.

Roles and responsibilities:

Contributors: SH, BS, IB, MB, DM, PR, had the study idea and designed the study. SS provided expertise to ensure implementation at the journal level was possible. MMS was responsible for statistical aspects, including the sample size calculation and the data analysis plan. BS and SH wrote the first draft of the study protocol. All authors critically revised the protocol and approved the final version.

Sponsor and contact information: Centre for Statistics in Medicine, Botnar Research Centre, University of Oxford, Windmill Road, Oxford OX3 7LD. Principal investigator: Benjamin Speich (Email: Benjamin.speich@ndorms.ox.ac.uk)

Sponsor and funders: The funders had no role in designing the study and will also have no role in conducting the study as well as in analysing and reporting study results.

Roles and responsibilities: The principal investigator (BS) is responsible for the preparation and the revisions of the study protocol, organising meetings of the steering committee, recruiting and randomizing eligible manuscripts as well as the publication of study reports. The steering committee (IB, MB, SH, DM, PR, BS, MMS, and SS) is in charge of participating in the elaboration of the protocol, defining and validating the additional short explanation for each CONSORT item, following the evolution of the committed study and for publishing the results of this study. MMS is responsible for the sample size calculation and the statistical analyses.

Table of content:

1. BACKGROUND AND RATIONAL ... 4
   1.1 Need for clinical research and epidemiologic transparency ... 4
   1.2 Transparency in published randomised controlled trials ... 4
   1.3 Journal attempts to improve reporting in published randomised controlled trials ... 5
2. HYPOTHESIS ... 6
3. OBJECTIVE ... 6
   3.1 Main objective ... 6
4. METHODS ... 7
   4.1 Trial design ... 7
   4.2 Study setting and eligibility criteria ... 7
   4.3 Interventions ... 8
   4.4 Outcomes ... 11
   4.5 Participant timeline ... 12
   4.6. Sample size ... 14
   4.7 Randomisation and blinding ... 15
   4.8 Statistical methods ... 16
      4.8.1 Populations of analysis ... 16
      4.8.2 Data analysis ... 17
      4.8.3 Analysis of primary endpoint ... 17
      4.8.4 Analysis of secondary endpoints ... 17
5 LEGAL AND GENERAL LOGISTICS ... 18
   5.1. Organisation of study ... 18
      5.1.1 Coordinating centre ... 18
      5.1.2 Scientific committee ... 18
   5.2. Regulatory aspects ... 19
6 PUBLICATION POLICY AND ACCESS TO DATA ... 20
REFERENCES ... 21
APPENDIX ... 24

1. Background and rational

1.1 Need for clinical research and epidemiologic transparency

There is substantial agreement that well conducted and reported randomised controlled trials (RCTs) generate the most trustworthy evidence when newly developed or already existing clinical interventions are evaluated (1-3). Besides the complexity and the high associated costs of conducting RCTs (4-6), there are major issues with their reporting that often make it difficult for researchers, clinicians, patients or policymakers to interpret the current evidence on a specific topic (7, 8). Chronologically, the most prominent difficulties in reporting consist of (i) poor reporting in study protocols for RCTs (9-12); (ii) a substantial fraction of trials are not registered, prematurely discontinued (most common due to difficulties with recruitment) and not published (13, 14); and (iii) that published RCTs are often poorly reported (7).

For clinicians, scientists and decision makers, published articles are often the only way to know how a study was conducted. In order to judge the internal and external validity of RCTs, it is crucial that these articles present transparent, accurate and unbiased information about the methods and conduct of the RCT.

1.2 Transparency in published randomised controlled trials

To improve the transparency in clinical and epidemiological research the international organisation called the EQUATOR (Enhancing the Quality and Transparency of Research) Network was founded in 2006 (15-20). This international network consists of researchers, epidemiologists, people in charge of recommendations for the presentation of articles or “reporting guidelines”, statisticians, clinicians and editors from some of the most prestigious journals (e.g., *Lancet, JAMA, Annals of Internal Medicine, BMJ*).

The CONSORT Statement (CONsolidated Standards for Reporting Trials), is perhaps the most important reporting guideline designed to help improve the transparency and quality of reporting of RCTs (21, 22). The CONSORT Statement, consisting of 25 items and a flow diagram which should be reported in papers describing RCTs. The last update of the CONSORT Statement was published simultaneously in 10 leading medical journals in 2010 (23). Currently CONSORT is endorsed by 585 journals (24). The CONSORT Statement guides authors, peer reviewers and journal editors on what information should be included in published reports of RCTs in order to facilitate critical judgment and interpretation of results. It is important to note, that adhering to the CONSORT Statement does not mean that the study is of high quality. However, reporting all items from the CONSORT list will enable readers to adequately judge the quality of RCTs.

A number of research studies have identified serious limitations in the reporting of published RCTs (3, 25-30). Despite some improvement in reporting following the implementation of the CONSORT Statement, there still remain major reporting deficiencies in published RCTs (31). For example, Odutayo and colleagues showed that a large proportion of RCTs published in December 2012 in PubMed did not define the primary outcome (31%), did not state the sample size calculation (45%) and did not explain the method of allocation concealment (50%) (32). This lack of transparency is a major limiting factor for the reader who assesses an article in order to find the answer to a specific question; it is also a major problem for scientists who perform systematic reviews and meta-analyses. Thus, some published trials may not be included in the meta-analysis because of their lack of transparency. Chan showed (25, 33) that 50% of efficacy outcomes and 65% of safety outcomes could not be included in meta-analyses because of how they were reported. Furthermore, even if these trials are included in systematic reviews and meta-analyses, an adequate risk of bias assessment is often not possible due to the poor reporting quality. Nevertheless, the main consequence of the lack of transparency is the risk of accepting treatments that are ineffective or cause serious adverse events (34).

1.3 Journal attempts to improve reporting in published randomised controlled trials

Journals can play a vital role in improving the reporting of published reports of RCTs. For example, a survey of authors' instructions on journal websites revealed that in 2014 63% (106 of 168) of biomedical journals mentioned CONSORT within their "Instructions to Authors" (35). Of those journals 38 (36%) required a CONSORT checklist as a condition of RCT report submission. Such implementation indicates some improvement over time compared to an assessment in 2007 when only 17 journals requested the CONSORT checklist (36). An interrupted time series analysis which assessed if the CONSORT for Abstracts guideline had an effect on the reporting quality, found that results are better reported in Journals which enforce the policy (37).

In a study published in 2016 authors of RCTs were asked by journal editors to use the web-based CONSORT tool at the manuscript revision stage (38). Authors who were randomly allocated to the intervention had access to a tool which allowed them to combine different CONSORT extensions (according to study design, medical field) to generate customised checklists. In the control group, authors had access to a CONSORT flow diagram generator. The goal was to improve the reporting of CONSORT items with a simple webtool. However, a quarter of all authors either wrongly selected a CONSORT extension or failed to select an extension, indicating that further education is needed in terms of when and how to implement CONSORT extensions.

A systematic scoping review conducted in 2017 by Blanco and colleagues summarised different interventions aimed to improve adherence to reporting guidelines (39) (manuscript with results currently under review. Draft received via personal communication). A number of different interventions were identified and some had also been tested at journals. However, the interventions, besides requesting submission of checklists from authors, required additional resources at the journal level (e.g. internal peer review by editorial assistants or inviting an additional statistical peer-reviewer (40, 41)). Therefore, it is unlikely that these interventions will be implemented in the vast majority of journals, especially not in smaller journals with limited resources. A study examining "the nature and extent of changes made to manuscripts after peer review, in relation to the reporting of methodological aspects of RCTs" and "the type of changes requested by peer reviewers" found that peer review did lead to some improvement in reporting (40).

Building on these findings we plan to evaluate the impact of inviting peer reviewers to explicitly use a short version of the CONSORT checklist (including a short explanation of those items) as part of their review process. If this intervention deems to be effective, it could be easily implemented by all medical journals without needing additional resources at a journal level.

2. Hypothesis

We propose an RCT to evaluate the impact of asking peer reviewers to use a short version of the CONSORT checklist when reviewing a manuscript of an RCT and whether it improves the completeness of reporting. Our hypothesis is that reminding peer reviewers of the CONSORT items (including a short explanation of those items) will result in higher adherence to CONSORT guidelines in published RCTs. We only selected a limited number of the CONSORT items because we did not want to deter peer reviewers with too much information. Since peer reviewing in general can be burdensome, we felt that this approach is more promising than listing all items, risking that the information will be ignored. The short version of the CONSORT checklist is based on the same items described in a previous study as the 10 most important and underreported CONSORT items (38).

3. Objective

3.1 Main objective

The main objective of this study is to evaluate the impact of asking peer reviewers during the standard peer-review process to ask them to use a short version of the CONSORT checklist (C-short) and whether it will improve the reporting in published RCTs compared to manuscripts where the peer reviewers underwent usual practice.

4. Methods

4.1 Trial design

This study is a multicentre RCT with articles being the unit of randomisation (Figure 1; allocation ratio 1:1). A multicentre parallel arm RCT with randomisation at the individual article level was chosen instead of a cluster RCT because the risk of any "contamination" on journal level is not given as the intervention will be implemented by an external researcher (i.e. BS). The possibility of contamination due to the possibility that peer reviewer are invited to assess several RCTs and are randomised into both arms was judged as relatively small and therefore we do not plan to adjust for clustering by journal. The journal staff (i.e. editors) will not be actively told which manuscript was allocated to the proposed intervention and which to the control group.

Figure 1: Study flowchart

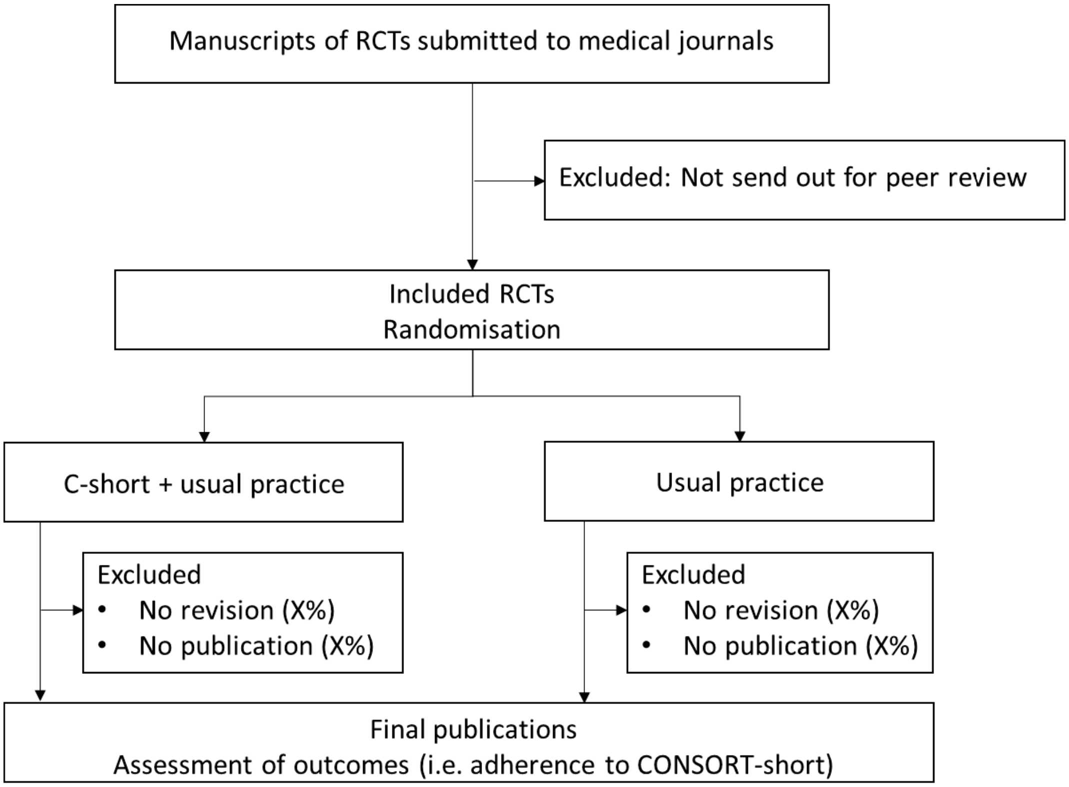

4.2 Study setting and eligibility criteria

The population will be defined on two levels. Included journals and included articles.

Included journals must: i) endorse the CONSORT Statement (e.g. assessed via journals Instruction to Authors); ii) publish primary results of at least five RCTs in 2017 (identified in a brief PubMed search as publishing RCTs in 2017). To be efficient, we plan to contact (via

email) the editors of eligible journals within a publishing house (i.e. journals which are part of the BMJ series, BMC series, PLoS, Lancet, JAMA) instead of separate journals. A description of the requirements for participation and a short summary information sheet will be included as part of the email invitation sent to journal editors. If a journal is eligible, and agrees to take part, the journal will also need to provide access to their journal editorial system (e.g. ScholarOne, Editorial Manager) to enable the external researcher (i.e. BS) to screen and randomise eligible manuscripts. In cases this is not possible, we will explore with separate journals if it would be possible to grant limited access (e.g. only rights to screen studies) and that the emails from the intervention would be sent by a person from the editorial team.

We will include all submitted manuscripts reporting RCTs for which the journal decides to send out for external peer review. Since the 10 chosen CONSORT checklist items are applicable to different study designs, we will include all RCTs regardless of study design (e.g. parallel group trial, cluster trial, superiority trial, non-inferiority trial). Articles presenting clearly secondary trial results, additional time points, economic analyses, or any other analyses derived from an RCT dataset not including the study's main results will be excluded. Furthermore, RCTs which are clearly labelled as a pilot or feasibility study or randomise animals or cells instead of individuals will be excluded.

Details of journal manuscript submission and peer review processes, including, consent and potential confidentiality issues will be discussed in detail with each journal by teleconference and/or face to face prior to the journal agreeing to take part to ensure that randomisation of manuscripts is feasible. We considered conducting randomisation at the level of the journal (i.e. cluster RCTs). However, in order to make the intervention as easy and simple to implement (and with little or no additional effort from the journal) we believe that randomisation at the manuscript level - with an external researcher implementing the intervention within the existing journal management systems - will be the most efficient study design.

In participating journals, the external investigator (BS) will have access to the editorial management software (e.g. ScholarOne or Editorial Manager) and will check at least twice a week (using automated report lists) all research manuscripts that are sent out for external peer review. As soon as the first peer-reviewer accepts the invitation to review, the manuscript will be randomised to the intervention or control arm (see "Randomisation" for more details). It is possible that this process might be slightly different amongst different included journals.

4.3 Interventions

Experimental group: C-short plus usual practice

After accepting to review an article, peer reviewers will receive the automated, journal specific standard email with general information as per each journal's usual practice (e.g. where to access the manuscript, date when the peer review report is due). In addition, peer-reviewers who received a manuscript which was randomised to C-short will receive an additional email including a short version of the CONSORT checklist (C-short) (either within the email or a as an attachment; based on the preferences and possibilities of the journal) focusing on the 10 most important and most poorly reported items (Table 1; as previously defined by a group of experts of the CONSORT Group (38)). Peer-reviewers will be asked to pay particular attention to items in the C-short checklist and request authors to report on these items, if not already adequately reported. This second email, containing the C-short checklist, is not generated automatically within the existing journal editorial management system (e.g. ScholarOne or Editorial Manager); it will be sent by the investigator who has access to the journal editorial system (BS). An example of this additional email is presented within the appendix (appendix 1; the exact wording might be changed according to the preferences of the participating journals). At least twice a week the editorial management system will be checked for each journal and if a peer reviewer has accepted an invitation to review, an email containing the C-short intervention will be generated and sent. It might be possible that some journals will only provide the right to access and read manuscripts in the editorial management system, but not to send emails. If this is the case, the corresponding Editor (or designated person within the journal) will be informed to send the emails.

Development and testing of the short explanation of the C-short items:

We chose the 10 most important and poorly reported CONSORT items as identified by a group of CONSORT experts in a previous study conducted by Hopewell and colleagues (38). The selection of the items was based on expert opinion and empirical evidence whenever available (38). In addition, we have added a short explanation for each of the 10 items. These short explanations were extracted and amended from the CONSORT explanation and elaboration paper (21) and from COBWEB which is online writing aid tool (42). The short explanation was discussed and adapted by the scientific committee.

Control group: Usual practice:

After accepting to review an article, peer reviewers will receive the automated, journal specific standard email with general information as per each journal's usual practice (e.g. where to access the manuscript, date until when the peer review report is due). However, they will not receive the second email, sent by the investigator who has access to the journal editorial system (BS) which contains the C-short checklist.

Table 1: The ten most important and poorly reported CONSORT items as defined by a group of experts on the CONSORT statement (38). For better understanding key features were summarised within a short explanation (extracted from the CONSORT explanation and elaboration paper (21) as well as from the COBWEB tool (42)).

Item	Section	CONSORT item	Short explanation
1	Outcomes (6a)	Completely defined pre-specified primary outcome measure, including how and when they were assessed	Is it clear (1) what the primary outcome is (usually the one used in the sample size calculation), (2) how it was measured (if relevant; e.g. which score used), (3) at what time point, and (4) what the analysis metric was (e.g. change from baseline, final value)?
2	Sample size (7a)	How sample size was determined	Is there a clear description of how the sample size was determined, including (1) the estimated outcomes in each group; (2) the α (type I) error level; (3) the statistical power (or the β (type II) error level); and (4) for continuous outcomes, the standard deviation of the measurements?
3	Sequence generation (8a)	Method used to generate random allocation sequence	Does the description make it clear if the "assigned intervention is determined by a chance process and cannot be predicted"?
4	Allocation concealment (9)	Mechanism used to implement random allocation sequence (such as sequentially numbered containers), describing any steps taken to conceal the sequence until interventions were assigned	Is it clear how the care provider enrolling participants was made ignorant of the next assignment in the sequence (different from blinding)? Possible methods can rely on centralised or "third-party" assignment (i.e., use of a central telephone randomisation system, automated assignment system, sealed containers).
5	Blinding (11a)	If done, who was blinded after assignment to interventions (for example, participants, care providers, those assessing outcomes)	Is it clear if (1) healthcare providers, (2) patients, and (3) outcome assessors are blinded to the intervention? General terms such as "double-blind" without further specifications should be avoided.
6	Outcomes and estimation (17a/b)	For the primary outcome, results for each group, and the estimated effect size and its precision (such as 95% confidence intervals)	Is the estimated effect size and its precision (such as standard deviation or 95% confidence intervals) for each treatment arm reported? When the primary outcome is binary, both the relative effect (risk ratio, relative risk) or odds ratio) and the absolute effect (risk difference) should be reported with confidence intervals.
7	Harms (19)	All-important harms or unintended effects in each group	Is the number of affected persons in each group, the severity grade (if relevant) and the absolute risk (e.g. frequency of incidence) reported? Are the number of serious, life threatening events and deaths reported? If no adverse event occurred this should be clearly stated.
8	Registration (23)	Registration number and name of trial registry	Is the registry and the registration number reported? If the trial was not registered, it should be explained why.
9	Protocol (24)	Where trial protocol can be accessed	Is it stated where the trial protocol can be assessed (e.g. published, supplementary file, repository, directly from author, confidential and therefore not available)?
10	Funding (25)	Sources of funding and other support (such as supply of drugs) and role of funders	Are (1) the funding sources, and (2) the role of the funder(s) described?

4.4 Outcomes

<u>Primary outcome:</u>

The primary outcome of this study will be the difference of the mean proportion of adequately reported items of the 10 most important and poorly reported CONSORT items between the two intervention arms.

<u>Secondary outcomes:</u>

Secondary outcomes will include the following:

- Mean proportion of adequate reporting of the 10 most important and poorly reported CONSORT items, considering each sub-item (see also "Assessment of outcomes") as a separate item.
- Mean proportion for each of the 10 most important and poorly reported CONSORT items separately (including also separate analysis of sub-items).
- Time from assigning an academic editor until the first decision (as communicated to the author after the first round of peer-review).
- Proportion of articles directly rejected after the first round of peer-review.
- Proportion of articles published.

<u>Additional outcomes:</u>

For journals where peer reviewer comments are subsequently published alongside the published article, we will examine the peer reviewer comments for any reference to CONSORT and trial reporting. We will contact those journals which do not make peer reviewer comments publicly available, to see if they still could be used for such an analyses under the condition that only anonymised data will be published.

<u>Data collection methods:</u>

The outcomes will be assessed independently by two (blinded or at least partially blinded; see "blinding") outcome assessors with expertise in the design and reporting of clinical trials. Any disagreement will be resolved by consensus or if necessary by consulting a third assessor. To ensure consistency between reviewers, we will first pilot the data extraction form; any disparities in the interpretation will be discussed and the data extraction form will be modified accordingly.

Adequate reporting of items will be assessed from published full-text publications adhering to the CONSORT C-short checklist (21). The following included items have sub-items which will be extracted separately:

- Outcomes (item 6a): (i) Define primary outcome, (ii) how it was measured, (iii) at what time point, and (iv) the analysis metric (e.g. change from baseline, final value).
- Sample size (item 7a): (i) The estimated outcomes in each group, (ii) the α (type I) error level, (iii) the statistical power (or the β (type II) error level), (iv) for continuous outcomes, the standard deviation of the measurements
- Blinding (item 11a): Is the blinding status clear for the following persons: (i) Healthcare provider, (ii) patients, and (iii) outcome assessors.
- Funding (item 25): (i) The funding source, and (ii) the role of funder in the design, conduct, analysis, and reporting.

All items will be judged as either "yes" meaning adequately reported, "no" meaning not adequately reported, or "NA" meaning that this sub-item is not applicable for this RCT. Items with different sub-items will only be judged as adequately reported if all relevant sub-items were adequately reported.

- Time from assigning an academic editor until the first decision: The day when the academic editor was assigned and the day of the first decision (e.g. major revision, minor revision, rejected) will be extracted to calculate the number of days until the first decision.
- Proportion of articles directly rejected after the first round of peer-review: Articles which were not invited for re-submission will be labelled and counted.
- Proportion of articles published: Articles which will be published will be counted and collected for data extraction.

The outcomes "time from assigning an academic editor until the first decision", "proportion of articles directly rejected after the first round of peer-review", and "proportion of articles published" will be extracted directly from editorial management software of the journal.

4.5 Participant timeline

The overview of the study schedule, including enrolment, intervention and assessments is presented in Table 2.

Table 2: Study schedule

	**Enrolment**	**Allocation and intervention**	**Intervention**	**Post-intervention**	
Time-point	*Studies which are sent out for peer-review*	*After first peer-reviewer accepts invitation*	*Whenever an additional peer-reviewer accepts invitation*	*First decision by journal*	*Published manuscripts*
Eligibility screen	X				
Allocation		X			
Intervention:					
C-short + usual care		X	X		
Usual care		X	X		
Assessment of trial characteristics:					
Funding source					X
Study centres (single centre or multicentre)					X
Sample size					X
Study design (e.g. parallel arm, crossover)					X
Hypothesis (e.g. superiority, non-inferiority)					X
Medical field					X
Intervention tested					X
Number of trial arms					X
Number of peer-reviewers					X
Journal which published the manuscript					X
Number of journals requesting CONSORT adherence (submission of checklist mandatory)					X
Assessment of outcomes:					
Time from assigning an academic editor until the first decision				X	
Proportion of articles directly rejected after the first round of peer-review				X	
Proportion of articles published					X
Adherence to CONSORT items and sub-items					X

4.6. Sample size

For the sample size calculation we hypothesise in a first scenario (Table 3) that the intervention C-Short will result in a 25% relative increase in adequate reporting compared to the control (meaning that 70% of items will be adequately reported in the intervention group and 56% in the control group). This is based on the rate of reporting of the 10 most important and poorly reported items was 0.56 (meaning that a mean of 56% of the 10 most important and poorly reported items were reported) in the control group of a previous study called WebCONSORT (38). The standard deviation (SD) in the same study was 0.23. However, we calculated our sample size to account for a slightly bigger variability in our data (SD = 0.25).To demonstrate a significant difference with a power of 90% and a type 1 error at 5% a total of 136 articles will be required in this scenario (68 per treatment arm; based on a two sided t-test).

The staff from one journal which will most likely be included (i.e. *PLoS One*) pointed out that 3 out of the 10 assessed items (i.e. item “Registration”, “Protocol”, and “Funding”) should always be implemented given their template. Assuming that this journal will recruit a high proportion, and that also other journals might update their templates, we increased the sample size in a second scenario, in which all these 3 items would have an overall adherence of 90% in the control arm (Table 3). This would entail an overall baseline adherence with the 10 CONSORT-short items of 71%. Based on a two sided t-test, a sample size of 166 (83 per treatment arm) will have a power of 80% to find a 15% relative increase (71% adherence in control group; 82% adherence in intervention group; SD = 0.25; a type 1 error at 5%).

Since the final sample size will be based on the number of articles published, rather than on the number of manuscripts randomised, eligible RCTs will be included and randomised until the number of 83 published RCTs is reached in each arm (resulting in no less than 166 articles), to avoid loss of power due to potential imbalance between arms. Recruitment will be stopped as soon as both arms reach the sample size of 83. After recruitment stop we will wait three month so that manuscripts which are still in production can be published. Manuscripts which are published after the three month period will be excluded.

Table 3: Assumptions for sample size calculations in two different scenarios.

Item	CONSORT item	Scenario 1. Adequate reporting as published in WebCONSORT	Scenario 2. Adapted from Scenario 1
1	Outcomes (6a)	77% (79 of 103)	77% (79 of 103)
2	Sample size (7a)	83% (85 of 103)	83% (85 of 103)
3	Sequence generation (8a)	76% (78 of 103)	76% (78 of 103)
4	Allocation concealment (9)	55% (57 of 103)	55% (57 of 103)
5	Blinding (11a)	35% (36 of 103)	35% (36 of 103)
6	Outcomes and estimation (17a	44% (45 of 103)	44% (45 of 103)
7	Harms (19)	71% (73 of 103)	71% (73 of 103)
8	Registration (23)	69% (71 of 103)	90%
9	Protocol (24)	19% (20 of 103)	90%
10	Funding (25)	34% (35 of 103)	90%
**Overall**		**56%**	**71%**

Abbreviation: CONSORT= CONsolidated Standards for Reporting Trials

4.7 Randomisation and blinding

Articles, which meet the eligibility criteria as a primary report of an RCT, for which the journal decides to send out for external peer review will be randomised into one of the two groups (allocation 1:1). The randomisation list will be created by the study-randomizer system (43) using random block sizes between 2 and 8 and stratification by journal. As soon as the first peer-reviewer accepts the invitation, the manuscript will be included and randomised to one of the two intervention arms. One of the investigators (BS) will log onto the study randomizer-system (43) entering the study identification number (ID; provided from the Journal), the study title, as well as the journal the study was submitted to. Subsequently, all additional peer-reviewers accepting the invitation to review the same manuscript will receive the same intervention (C-short plus usual practice or usual practice only) as the first peer-reviewer.

Authors will be blinded to the intervention allocation. Editors will not be actively informed about the randomisation (possible exception listed under “4.3 Interventions”). To avoid potential bias, peer reviewers and manuscript authors will not be informed of the study hypothesis, design and intervention.

Outcomes will be assessed in duplicate (see assessment of outcomes). At least one outcome assessors will be blinded. Due to restricted resources it might be possible that the investigator conducting the randomisation (BS) will be included in the data-extraction from published manuscripts.

4.7 Data management and confidentiality

Outcomes from publications will be assessed and extracted in duplicate. Since this information is not confidential, we will use Google Forms for data extraction from published RCTs. Data entered will be validated for completeness.

Data from the editorial manager software (e.g. Title of manuscript, first author, randomisation ID, Journal, date when manuscript was accepted by and academic editor, date when the final decision was made, final decision, number of peer-reviewers who peer reviewed the manuscript, the peer review) will be extracted, anonymised and entered in a password protected database which is saved on a server from the University of Oxford. Data will be managed and curated according to University of Oxford regulations, which includes regular back-up (on a daily basis) of the virtual drives where the data are stored.

The raw data extracted from the included manuscripts can be made openly accessible in an anonymised way (i.e. giving the included RCT a number instead of identifying them). Derived/aggregated data, including anonymised information generated from the journals' editorial manager software, will be stored and made available to the research community when the project ends (see also "8. Publication policy and access to data"). Where appropriate, the researcher who has access to the editorial manager software (BS) and anyone else who will see the identifiable data will sign a confidentially agreement with the participating journals, confirming that they will not share identifiable data with any other party. Journals such as the BMJ series state in their Company Privacy Statement that research programmes for quality improvement might be in place. Furthermore, peer reviewers for all BMJ journals receive the following statement in their invitation letter "*We are constantly trying to find ways of improving the peer review system and have an ongoing programme of research. If you do not wish your review entered into a study please let us know by emailing […] as soon as possible*."

4.8 Statistical methods

4.8.1 Populations of analysis

The main population for analysis will be all manuscripts randomised and accepted for publication in the participating journals. Differently from RCTs conducted with patients, where drop outs need to be carefully considered (e.g. multiple imputation of missing data), we are only interested in the reporting adherence of RCTs that are published. All outcomes will be calculated based on the main population for analysis. The secondary outcome "Time to the first decision", will additionally be calculated considering all randomised manuscripts (including the ones which were not published).

4.8.2 Data analysis

All quantitative variables will be described using means and standard deviations, or median and interquartile ranges in case severe departures from a normal distribution are identified. Data distribution will be inspected visually (i.e. by histograms) instead of performing formal statistical tests for normality. Categorical variables will be described using frequencies and percentages. For the primary and secondary outcomes, we will estimate the difference between means between the two groups and report them with respective 95% confidence intervals.

4.8.3 Analysis of primary endpoint

The primary outcome will be the difference of the mean proportion of adequately reported items of the 10 most important and poorly reported CONSORT items. If the data on the primary outcome is normally distributed then the two groups (i.e. C-short plus usual practice vs. usual practice) will be compared using an unpaired Student's t-test to compare the unadjusted mean proportion of adequate reporting. If the data is not normally distributed, comparisons will be performed using a non-parametric equivalent test (i.e. Wilcoxon-Mann-Whitney test for testing whether the population medians of the two groups are the same).

For the analyses of the primary outcomes a p-value of 0.05 (5% significance level) will be used to indicate statistical significance and treatment effect (mean difference) reported with 95% confidence intervals (or median and respective interquartile ranges, in case of asymmetric distribution). Exact p-values will be presented up to three decimal places. We anticipate there will be no missing data in this study, neither at the individual C-short items, nor at the manuscript level. This is due to the study design, which will include only the randomised manuscripts that are accepted for publication.

4.8.4 Analysis of secondary endpoints

To investigate the effect of the intervention on the secondary outcomes, mean differences with respective 95% confidence intervals will also be reported for these outcomes. If normality is not observed for any of the continuous secondary outcomes, the same strategy adopted for the primary outcome (use of a non-parametric equivalent to the Student's *t*-test) will be used.

A p-value of 0.05 will indicate statistical significance for the observed treatment effect on the secondary outcomes. Exact p-values will be presented up to three decimal places. Similarly to the primary outcome, we anticipate there will be no missing data for any of the secondary outcomes, as we will have access to the Editorial Management system of the included journals, where all relevant information is automatically reported.

4.8.5 Pre-specified subgroup analysis

No formal subgroup comparative analysis is planned for the primary or secondary outcomes. However, the effect of the intervention on the primary outcome within subgroups, will be presented using forest plots to visually examine whether it differs according to some variables, such as: (1) Journals that actively implement the CONSORT Statement (defined as requiring authors to submit a completed CONSORT checklist alongside their manuscript) vs. journals that are not actively implementing the CONSORT Statement; (2) sample size (n < 100 vs. n ≥ 100); and (3) impact factor (<5, 5.1-10; >10) as there is evidence that higher impact factor as well as higher sample size are associated with higher adherence to reporting guidelines (44). These analyses will be exploratory, with the aim of supporting new hypothesis generation, rather than conclusive.

5 Legal and general logistics

5.1. Organisation of study

5.1.1 Coordinating centre

The coordinating centre's, will be the Centre for Statistics in Medicine at the University of Oxford under the responsibilities of Dr Sally Hopewell and Dr Benjamin Speich.

The coordinating centre's will ensure the following missions:

- Training of the staff
- Implementation of quality control
- Logical controls of data
- Follow-up on requests for correction/validation
- Statistical analysis
- Archiving of data

5.1.2 Scientific committee

The scientific committee is composed of:

- Prof Isabelle Boutron: Centre D'Épidémiologie Clinique Hôtel-Dieu, Paris Descartes University, France
- Prof Matthias Briel, University of Basel, Switzerland
- Associate Prof Sally Hopewell: Centre for Statistics in Medicine, University of Oxford, UK
- Prof David Moher: Centre for Journalology, Clinical Epidemiology Program, Ottawa Hospital Research Institute, Canada

- Prof Philippe Ravaud: Centre d’Épidémiologie Clinique Hôtel-Dieu, Paris Descartes University, France
- Dr Benjamin Speich, Centre for Statistics in Medicine, University of Oxford, UK
- Dr. Michael M Schlussel, Centre for Statistics in Medicine, University of Oxford, UK
- Dr Sara Schroter, The BMJ, London, UK

The scientific committee is in charge of:

- Participating in the elaboration of the protocol
- Defining and validating the additional short explanation for each CONSORT item.
- Following the evolution of the committed study
- Publishing the results of this study

5.2. Regulatory aspects

Ethical approval for this study will be sought from the Central University Research Ethics Committee (CUREC) of the University of Oxford. Any amendments in the conduct of the study, collection of outcomes or analysis will be reported to the CUREC. The tested intervention has the goal to improve the quality of published journals (i.e. the adherence to CONSORT) and could also be implemented as usual practice without testing at the journal level. In agreement with another study, testing a similar intervention (45), we think that it is ethical to conduct this study without obtaining written consent. The main reason for this procedure are the following:

- Informing the authors and peer-reviewers would make it impossible to measure the effect of our intervention. In short, informing peer-reviewers and authors would create an artificial context which would not be comparable any more to the “real world context”. Authors and peer-reviewers would most likely be much more aware of CONSORT if they received information about the study. Furthermore, being aware to participate in a study could strongly influence the natural behaviour of peer-reviewers (e.g. putting more effort into reviewing a manuscript than under “real world conditions”) but also of authors.
- The intervention does not pose any risk of harms for authors and peer-reviewers.
- The intervention is not a medical intervention but rather tries to improve the research quality and journal processes.
- Several journal series (e.g. BMJ series) have Company Privacy Statements in place which clearly mention that research programmes might be in place for quality improvement.
- The intervention could be part of the routine at any Journal without previous assessment of its efficacy.

- No data which identifies participating manuscripts will be published.

6 Publication policy and access to data

The results from this study will be published in a peer-reviewed journal irrespective of the study results. Authorship to publications will be granted according to the rules of the International Committee of Medical Journal Editors (ICMJE). We plan to publish the full anonymised dataset as a supplementary file together with the main publication.

References

1. Duley L, Antman K, Arena J, Avezum A, Blumenthal M, Bosch J, et al. Specific barriers to the conduct of randomized trials. Clin Trials. 2008;5(1):40-8.
2. Collins R, MacMahon S. Reliable assessment of the effects of treatment on mortality and major morbidity, I: clinical trials. Lancet. 2001;357(9253):373-80.
3. Chalmers I. Unbiased, relevant, and reliable assessments in health care: important progress during the past century, but plenty of scope for doing better. BMJ. 1998;317(7167):1167-8.
4. Collier R. Rapidly rising clinical trial costs worry researchers. CMAJ. 2009;180(3):277-8.
5. Eisenstein EL. Commentary on Sertkaya et al. and Larson et al. Clin Trials. 2016;13(2):137-9.
6. Sertkaya A, Wong HH, Jessup A, Beleche T. Key cost drivers of pharmaceutical clinical trials in the United States. Clin Trials. 2016;13(2):117-26.
7. Glasziou P, Altman DG, Bossuyt P, Boutron I, Clarke M, Julious S, et al. Reducing waste from incomplete or unusable reports of biomedical research. Lancet. 2014;383(9913):267-76.
8. Chan AW, Song F, Vickers A, Jefferson T, Dickersin K, Gotzsche PC, et al. Increasing value and reducing waste: addressing inaccessible research. Lancet. 2014;383(9913):257-66.
9. Chan AW, Tetzlaff JM, Altman DG, Dickersin K, Moher D. SPIRIT 2013: new guidance for content of clinical trial protocols. Lancet. 2013;381(9861):91-2.
10. Chan AW, Tetzlaff JM, Altman DG, Laupacis A, Gotzsche PC, Krleza-Jeric K, et al. SPIRIT 2013 statement: defining standard protocol items for clinical trials. Ann Intern Med. 2013;158(3):200-7.
11. Chan AW, Tetzlaff JM, Gotzsche PC, Altman DG, Mann H, Berlin JA, et al. SPIRIT 2013 explanation and elaboration: guidance for protocols of clinical trials. BMJ. 2013;346:e7586.
12. Lund H, Brunnhuber K, Juhl C, Robinson K, Leenaars M, Dorch BF, et al. Towards evidence based research. BMJ. 2016;355:i5440.
13. Kasenda B, von Elm E, You J, Blumle A, Tomonaga Y, Saccilotto R, et al. Prevalence, characteristics, and publication of discontinued randomized trials. JAMA. 2014;311(10):1045-51.
14. Alturki R, Schandelmaier S, Olu KK, von Niederhausern B, Agarwal A, Frei R, et al. Premature trial discontinuation often not accurately reflected in registries: comparison of registry records with publications. J Clin Epidemiol. 2017;81:56-63.
15. Altman DG, Simera I, Hoey J, Moher D, Schulz K. EQUATOR: reporting guidelines for health research. Lancet. 2008;371(9619):1149-50.
16. Simera I. EQUATOR Network collates resources for good research. BMJ. 2008;337:a2471.
17. Simera I, Altman DG. Writing a research article that is "fit for purpose": EQUATOR Network and reporting guidelines. Evid Based Med. 2009;14(5):132-4.
18. Simera I, Altman DG, Moher D, Schulz KF, Hoey J. Guidelines for reporting health research: the EQUATOR network's survey of guideline authors. PLoS Med. 2008;5(6):e139.
19. Simera I, Moher D, Hirst A, Hoey J, Schulz KF, Altman DG. Transparent and accurate reporting increases reliability, utility, and impact of your research: reporting guidelines and the EQUATOR Network. BMC Med. 2010;8:24.
20. Simera I, Moher D, Hoey J, Schulz KF, Altman DG. The EQUATOR Network and reporting guidelines: Helping to achieve high standards in reporting health research studies. Maturitas. 2009;63(1):4-6.
21. Moher D, Hopewell S, Schulz KF, Montori V, Gotzsche PC, Devereaux PJ, et al. CONSORT 2010 explanation and elaboration: updated guidelines for reporting parallel group randomised trials. BMJ. 2010;340:c869.

22. Schulz KF, Altman DG, Moher D, Group C. CONSORT 2010 statement: updated guidelines for reporting parallel group randomised trials. BMJ. 2010;340:c332.
23. Schulz KF, Altman DG, Moher D, Group C. CONSORT 2010 Statement: updated guidelines for reporting parallel group randomised trials. BMC Med. 2010;8:18.
24. CONSORT Transparent Reporting of Trials. Endorsers. Journals and Organizations. http://www.consort-statement.org/about-consort/endorsers [accessed: 27 February 2019]
25. Chan AW, Hrobjartsson A, Haahr MT, Gotzsche PC, Altman DG. Empirical evidence for selective reporting of outcomes in randomized trials: comparison of protocols to published articles. JAMA. 2004;291(20):2457-65.
26. Chalmers I, Glasziou P. Avoidable waste in the production and reporting of research evidence. Lancet. 2009;374(9683):86-9.
27. Ioannidis JP, Lau J. Completeness of safety reporting in randomized trials: an evaluation of 7 medical areas. JAMA. 2001;285(4):437-43.
28. Glasziou P, Meats E, Heneghan C, Shepperd S. What is missing from descriptions of treatment in trials and reviews? BMJ. 2008;336(7659):1472-4.
29. Boutron I, Dutton S, Ravaud P, Altman DG. Reporting and interpretation of randomized controlled trials with statistically nonsignificant results for primary outcomes. JAMA. 2010;303(20):2058-64.
30. Hopewell S, Dutton S, Yu LM, Chan AW, Altman DG. The quality of reports of randomised trials in 2000 and 2006: comparative study of articles indexed in PubMed. BMJ. 2010;340:c723.
31. Turner L, Shamseer L, Altman DG, Schulz KF, Moher D. Does use of the CONSORT Statement impact the completeness of reporting of randomised controlled trials published in medical journals? A Cochrane review. Syst Rev. 2012;1:60.
32. Odutayo A, Emdin CA, Hsiao AJ, Shakir M, Copsey B, Dutton S, et al. Association between trial registration and positive study findings: cross sectional study (Epidemiological Study of Randomized Trials-ESORT). BMJ. 2017;356:j917.
33. Chan AW, Krleza-Jeric K, Schmid I, Altman DG. Outcome reporting bias in randomized trials funded by the Canadian Institutes of Health Research. CMAJ. 2004;171(7):735-40.
34. Glass KC. Toward a duty to report clinical trials accurately: the clinical alert and beyond. J Law Med Ethics. 1994;22(4):327-38.
35. Shamseer L, Hopewell S, Altman DG, Moher D, Schulz KF. Update on the endorsement of CONSORT by high impact factor journals: a survey of journal "Instructions to Authors" in 2014. Trials. 2016;17(1):301.
36. Hopewell S, Altman DG, Moher D, Schulz KF. Endorsement of the CONSORT Statement by high impact factor medical journals: a survey of journal editors and journal 'Instructions to Authors'. Trials. 2008;9:20.
37. Hopewell S, Ravaud P, Baron G, Boutron I. Effect of editors' implementation of CONSORT guidelines on the reporting of abstracts in high impact medical journals: interrupted time series analysis. BMJ. 2012;344:e4178.
38. Hopewell S, Boutron I, Altman DG, Barbour G, Moher D, Montori V, et al. Impact of a web-based tool (WebCONSORT) to improve the reporting of randomised trials: results of a randomised controlled trial. BMC Med. 2016;14(1):199.
39. Blanco D, Kirkham JJ, Altman DG, Moher D, Boutron I, Cobo E. Interventions to improve adherence to reporting guidelines in health research: a scoping review protocol. BMJ Open. 2017;7(11):e017551.
40. Hopewell S, Collins GS, Boutron I, Yu LM, Cook J, Shanyinde M, et al. Impact of peer review on reports of randomised trials published in open peer review journals: retrospective before and after study. BMJ. 2014;349:g4145.
41. Cobo E, Selva-O'Callagham A, Ribera JM, Cardellach F, Dominguez R, Vilardell M. Statistical reviewers improve reporting in biomedical articles: a randomized trial. PLoS One. 2007;2(3):e332.
42. Barnes C, Boutron I, Giraudeau B, Porcher R, Altman DG, Ravaud P. Impact of an online writing aid tool for writing a randomized trial report: the COBWEB (Consort-based WEB tool) randomized controlled trial. BMC Med. 2015;13:221.

43. Study Randomizer. https://studyrandomizer.com/ [accessed: 23. January 2019].
44. Jin Y, Sanger N, Shams I, Luo C, Shahid H, Li G, et al. Does the medical literature remain inadequately described despite having reporting guidelines for 21 years? - A systematic review of reviews: an update. Journal of multidisciplinary healthcare. 2018;11:495-510.
45. Hair K, Macleod MR, Sena ES. A randomised controlled trial of an Intervention to Improve Compliance with the ARRIVE guidelines (IICARus). bioRxiv. 2018.

Appendix

Appendix 1: Example of the email which will be sent out in the intervention arm (C-Short). The exact wording might be slightly adapted according to the journal preferences.

Dear **Title, Name*,

We thank you for accepting to peer-review a manuscript for **journal name**. As we are trying to improve the reporting for randomised controlled trials according to the CONSORT guidelines, we would like to ask if you could check whether the following most important and poorly reported items are adequately implemented as indicated in the *table below/attached table*.

Item	Section	CONSORT item	Short explanation
1	Outcomes (6a)	Completely defined pre-specified primary outcome measure, including how and when they were assessed	Is it clear (1) what the primary outcome is (usually the one used in the sample size calculation), (2) how it was measured (if relevant; e.g. which score used), (3) at what time point, and (4) what the analysis metric was (e.g. change from baseline, final value)?
2	Sample size (7a)	How sample size was determined	Is there a clear description of how the sample size was determined, including (1) the estimated outcomes in each group; (2) the α (type I) error level; (3) the statistical power (or the β (type II) error level); and (4) for continuous outcomes, the standard deviation of the measurements?
3	Sequence generation (8a)	Method used to generate random allocation sequence	Does the description make it clear if the "assigned intervention is determined by a chance process and cannot be predicted"?
4	Allocation concealment (9)	Mechanism used to implement random allocation sequence (such as sequentially numbered containers), describing any steps taken to conceal the sequence until interventions were assigned	Is it clear how the care provider enrolling participants was made ignorant of the next assignment in the sequence (different from blinding)? Possible methods can rely on centralised or "third-party" assignment (i.e., use of a central telephone randomisation system, automated assignment system, sealed containers).
5	Blinding (11a)	If done, who was blinded after assignment to interventions (for example, participants, care providers, those assessing outcomes)	Is it clear if (1) healthcare providers, (2) patients, and (3) outcome assessors are blinded to the intervention? General terms such as "double-blind" without further specifications should be avoided.
6	Outcomes and estimation (17a/b)	For the primary outcome, results for each group, and the estimated effect size and its precision (such as 95% confidence intervals)	Is the estimated effect size and its precision (such as standard deviation or 95% confidence intervals) for each treatment arm reported? When the primary outcome is binary, both the relative effect (risk ratio, relative risk) or odds ratio) and the absolute effect (risk difference) should be reported with confidence intervals.
7	Harms (19)	All-important harms or unintended effects in each group	Is the number of affected persons in each group, the severity grade (if relevant) and the absolute risk (e.g. frequency of incidence) reported? Are the number of serious, life threatening events and deaths reported? If no adverse event occurred this should be clearly stated.
8	Registration (23)	Registration number and name of trial registry	Is the registry and the registration number reported? If the trial was not registered, it should be explained why.
9	Protocol (24)	Where trial protocol can be accessed	Is it stated where the trial protocol can be assessed (e.g. published, supplementary file, repository, directly from author, confidential and therefore not available)?
10	Funding (25)	Sources of funding and other support (such as supply of drugs) and role of funders	Are (1) the funding sources, and (2) the role of funder(s) described?

Your efforts are highly appreciated.

Kind regards,

journal name-Team
