# Supplementary material for: Reminding Peer Reviewers of Reporting Guideline Items to Improve Completeness in Published Articles: Primary Results of 2 Randomized Trials
Source: JAMA Netw Open. 2023 Jun 9;6(6):e2317651. doi: 10.1001/jamanetworkopen.2023.17651 (PMC10257091; doi:10.1001/jamanetworkopen.2023.17651)
Supplement: Supplement 2. — SPIRIT-PR Study Protocol [file jamanetwopen-e2317651-s002.pdf]

Impact of a short form of the SPIRIT checklist for peer reviewers to improve the reporting of protocols for randomised controlled trials published in biomedical journals: a randomised controlled trial

Short title: SPIRIT for Peer Review (SPIRIT-PR)

Principal investigator: Dr Benjamin Speich
Centre for Statistics in Medicine, University of Oxford

Dr Benjamin Speich
Centre for Statistics in Medicine,
Botnar Research Centre, University of Oxford, Windmill Road, Oxford OX3 7LD
Email: Benjamin.speich@ndorms.ox.ac.uk

SCIENTIFIC COMMITTEE

Prof Sally Hopewell, Centre for Statistics in Medicine, Oxford University, UK
Dr Sara Schroter, The BMJ, London, UK
Prof Matthias Briel, University of Basel, Switzerland
Dr Ayodele Odutayo, St Michael's Hospital, Toronto, Canada
Prof An-Wen Chan, University of Toronto, Canada
Dr Benjamin Speich, Centre for Statistics in Medicine, University of Oxford, UK
Dr Michael M Schlussel, Centre for Statistics in Medicine, University of Oxford, UK

Trial registration: This trial will be prospectively registered under the Open Science Framework. A registration in clinical trial registries such as clinicaltrials.gov will not be possible, because this study will not measure a health outcome in individuals.

Protocol version: Version 1.0.3 2020-05-20

Sponsor and Funding: Benjamin Speich is supported by an Advanced Postdoc.Mobility grant from the Swiss National Science Foundation (P300PB_177933). The funders had no role in designing the study and will also have no role in conducting the study or analysing and reporting study results.

Roles and responsibilities:

Contributors: SH, BS, and SS, had the study idea and designed the study. SS provided expertise to ensure implementation at the journal level was possible. SH, AO, BS, MB, AWC were involved in selecting the 10 SPIRIT items. MMS was responsible for statistical aspects, including the sample size calculation and the data analysis plan. BS and SH wrote the first draft of the study protocol. All authors critically revised the protocol and approved the final version.

Sponsor and contact information: Centre for Statistics in Medicine, Botnar Research Centre, University of Oxford, Windmill Road, Oxford OX3 7LD. Principal investigator: Benjamin Speich (Email: Benjamin.speich@ndorms.ox.ac.uk)

Roles and responsibilities: The principal investigator (BS) is responsible for the preparation and the revisions of the study protocol, organising meetings of the steering committee, recruiting and randomising eligible manuscripts as well as the publication of study reports. The steering committee (SH, SS, AWC, MB, BS, AO, and MMS) is in charge of participating in the elaboration of the protocol, defining and validating the key features for each SPIRIT item, following the evolution of the committed study and for publishing the results of this study. MMS is responsible for the sample size calculation and the statistical analyses.

Table of content:

1. Background and rational ... 4
1.1 Need for clinical research and epidemiologic transparency ... 4
1.2 Transparency in study protocols for randomised controlled trials ... 4
1.3 Journal attempts to improve reporting in published articles ... 5
2. Hypothesis ... 6
3. Objective ... 6
3.1 Main objective ... 6
4. Methods ... 6
4.1 Trial design ... 6
4.2 Study setting and eligibility criteria ... 7
4.4 Interventions ... 8
4.5 Outcomes ... 11
4.6 Participant timeline ... 13
4.7. Sample size ... 15
4.8 Randomisation and blinding ... 15
4.10 Statistical methods ... 16
4.10.1 Populations of analysis ... 16
4.10.2 Data analysis ... 17
4.10.3 Analysis of primary endpoint ... 17
4.10.4 Analysis of secondary endpoints ... 17
5 Legal and general logistics ... 18
5.1. Organisation of study ... 18
5.1.1 Coordinating centre ... 18
5.1.2 Scientific committee ... 18
5.2. Regulatory aspects ... 19
6 Publication policy and access to data ... 19
References ... 20
Appendix ... 22

1. Background and rational

1.1 Need for clinical research and epidemiologic transparency

For clinicians, scientists and decision makers, published articles are often the only way to know how a study was conducted. In order to judge the internal and external validity of a study, it is crucial that these articles present transparent, accurate and unbiased information about the methods and conduct of the study. To improve the transparency in clinical and epidemiological research the international organisation called the EQUATOR (Enhancing the Quality and Transparency of Research) Network was founded in 2006 (1-6). This international network consists of researchers, epidemiologists, methodologists, statisticians, clinicians and editors from some of the most prestigious journals (e.g., *Lancet, JAMA, Annals of Internal Medicine, BMJ*).

The EQUATOR network hosts a library of reporting guidelines, which are structured tools to help researchers to write manuscripts transparently (7). They provide a list of information which should be reported to make sure that the study can be correctly interpreted by researchers, clinicians, patients or policy makers. Despite large efforts in improving the reporting in scientific studies since the introduction of the CONSORT (Consolidated Standards of Reporting Trials) statement in 1996, reporting remains suboptimal (8).

1.2 Transparency in study protocols for randomised controlled trials

There is substantial agreement that well conducted and reported randomised controlled trials (RCTs) generate the most trustworthy evidence when clinical interventions are evaluated (9-11). To facilitate the conduct of a study and to pre-specify all relevant working steps, it is important that high quality study protocols are developed before the start of a RCT. Study protocols guide personnel involved in running a study and are also key documents when applying for ethical approval. Incomplete or unclear protocols can have serious implications for involved patients, trial staff, and sponsors. Missing key elements in trial protocols can lead to unreliable data which compromises the validity of trial results (12). In 2013 the reporting guideline for study protocols of clinical trials called SPIRIT (Standard Protocol Items: Recommendations for Interventional Trials) Statement was published (13, 14). The SPIRIT statement includes a checklist of 33 items as well as a diagram which should be reported in any trial protocol as a minimum standard for transparently reporting how a study will be conducted. Study protocols which are approved by ethical committees are usually confidential (15). However, since 2006 study protocols (usually shorter versions of the original study protocol) are also published as peer reviewed journal articles, which is a way of transparently sharing the study protocol that has become more popular in recent years (16). In addition, several leading medical journal (e.g. The BMJ; Lancet; New England Journal of Medicine;

JAMA; Annals of Internal Medicine) started to publish trial protocols alongside the trial publication of the main results (17).

A number of research studies have identified serious limitations in the reporting of published studies, independent of study design and use of the corresponding reporting guideline (8). However, according to our knowledge, adherence to SPIRIT has only been evaluated and published with study protocols from the NIHR HTA programme database (18). Kyte and colleagues found that some of the SPIRIT items were very poorly reported (e.g. allocation implementation: 34.7%; access to data: 2.7%). Our own research group is currently assessing reporting quality in study protocols that received ethical approval in the UK, Switzerland, Germany and Canada (*manuscript for protocol in preparation*). Our preliminary results for the ASPIRE (Adherence to SPIRIT) project show that there is also considerable room for improvement in the quality of reporting of study protocols approved by ethical review boards. This lack of transparency is a major limiting factor for the protocol readers to find out how the RCT was/will be conducted.

1.3 Journal attempts to improve reporting in published articles

Journals can play a vital role in improving the reporting of published studies. For example, a review of “Instructions to Authors” on biomedical journal websites revealed that in 2014 63% (106 of 168) of journals mentioned CONSORT, the most well-known reporting guideline (19). Of those journals 38 (36%) required a CONSORT checklist as a condition of RCT report submission. Such implementation indicates some improvement over time compared to an assessment in 2007 when only 17 journals requested the CONSORT checklist (20). An interrupted time series analysis which assessed if the CONSORT for Abstracts guideline had an effect on reporting quality, found that results are better reported in journals that requested the checklist (21).

A systematic scoping review conducted in 2017 by Blanco and colleagues summarised different interventions aimed to improve adherence to reporting guidelines (22, 23). A number of different interventions were identified and some had also been evaluated at journals. However, all the interventions, except requesting submission of checklists from authors, required additional resources at the journal level (e.g. internal peer review by editorial assistants or inviting an additional statistical peer reviewer (24, 25)). Therefore, it is unlikely that these interventions will be implemented in the majority of journals, especially not in smaller journals with limited resources. A study examining “the nature and extent of changes made to manuscripts after peer review, in relation to the reporting of methodological aspects of RCTs”

and “the type of changes requested by peer reviewers” found that peer review did lead to some improvement in reporting (24).

Building on these findings we are conducting a trial to evaluate the impact of inviting peer reviewers to explicitly use a short version of the CONSORT checklist (including a short explanation of those items) as part of the review process at a number of journals (26). In this document we describe the study protocol for a new trial assessing the impact of inviting peer reviewers to use a short version of the SPIRIT checklist when evaluating a study protocol submitted to BMJ Open (a large general medical journal that publishes many study protocols). If this intervention is shown to be effective, it could easily be implemented by medical journals without needing additional resources at a journal level.

2. Hypothesis

We propose an RCT to evaluate the impact of asking peer reviewers, reviewing a manuscript of a study protocol for an RCT, to use a short version of the SPIRIT checklist on the completeness of reporting. Our hypothesis is that reminding peer reviewers of the SPIRIT items (including a list of key features of those items) will result in higher adherence to SPIRIT guidelines in published RCT study protocols. We only selected a limited number of the SPIRIT items because we did not want to over burden peer reviewers since peer reviewing in general can already be burdensome (27). Including all 33 items would, in our opinion, pose a high risk that all of the information would be ignored. The development of the short version of this checklist is described under “4.4. Identifying the key features of the S-short items”.

3. Objective

3.1 Main objective

The main objective of this study is to evaluate the impact of additionally asking peer reviewers during the usual peer review process to use a short version of the SPIRIT checklist (S-short) on the reporting quality of published RCT protocols compared to usual peer review practice.

4. Methods

4.1 Trial design

This study is a single-centre parallel-arm superiority RCT with articles as the unit of randomisation (Figure 1; allocation ratio 1:1). The journal staff (i.e. editors) will not be told which manuscript are allocated to the intervention and which to the control group, but they will have access to all email communications to peer reviewers on the manuscript tracking system and therefore could identify manuscripts in the intervention arm.

Figure 1: Study flowchart

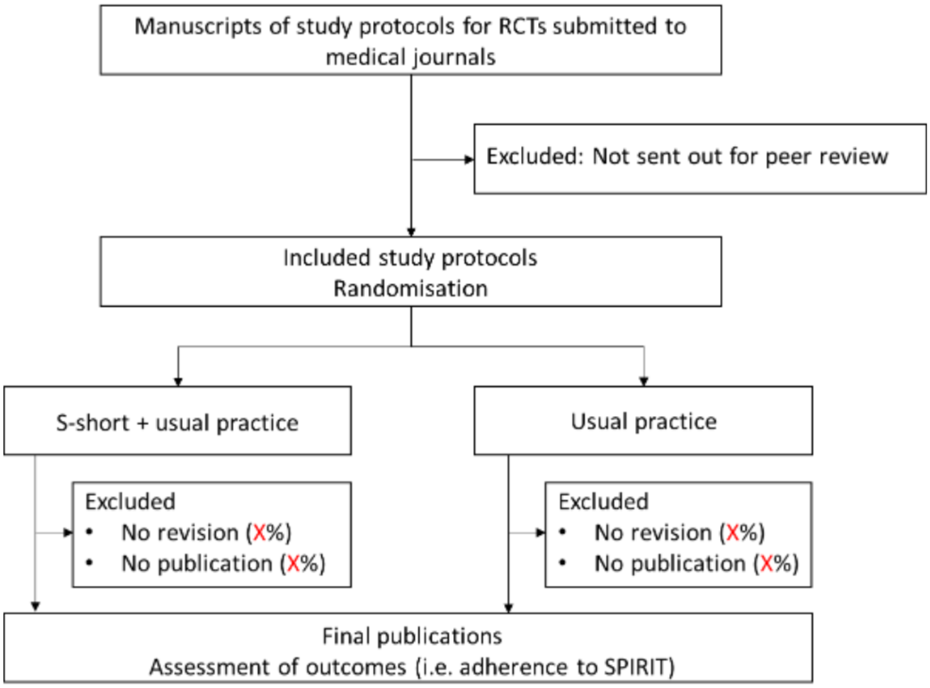

4.2 Study setting

This study will be conducted in collaboration with the journal BMJ Open. We searched PubMed and found that BMJ Open publishes approximately 40 eligible study protocols per month (see eligibility criteria below). Logistic and legal requirements are already set up in the frame of a current parallel ongoing trial (26). Under a confidentiality agreement, as part of the journal's quality improvement programme, BMJ Open has provided the principal investigator (BS) access to its editorial management software (ScholarOne) to screen and randomise eligible manuscripts and send requests to peer reviewers.

4.3. Eligibility criteria

We will include all submitted manuscripts reporting protocols for RCTs for which the journal decides to send out for external peer review. Since the 10 chosen SPIRIT checklist items are applicable to different study designs, we will include all RCTs regardless of study design (e.g. parallel group trial, cluster trial, superiority trial, non-inferiority trial). RCTs which are clearly labelled as a pilot or feasibility study or which randomise animals or cells instead of individuals will be excluded. Furthermore, separate data analysis plans will be excluded.

The external investigator (BS) will have access to the editorial management software. In addition, automated report lists will be used to identify all protocols of RCTs that are sent out for external peer review. As soon as the first peer reviewer accepts the invitation to review, the manuscript will be randomised to the intervention or control arm (see "Randomisation" for more details).

4.4 Interventions

Experimental group: S-short plus usual practice

After accepting to review an article, peer reviewers will receive the automated standard email with general information as usual practice (e.g. where to access the manuscript, date when the peer review report is due). In addition, peer reviewers who received a manuscript randomised to the intervention group will receive an additional email including a short version of the SPIRIT checklist (S-short) within the email and as an attachment focusing on the 10 most important and most poorly reported items (Table 1; as defined by the steering committee). Peer reviewers will be asked to pay particular attention to items in the S-short checklist and to request authors to report on these items, if not already adequately reported. This additional email (see Appendix 1 for example), containing the S-short checklist, is not generated automatically by ScholarOne but will be sent manually on the platform by BS, appearing to come from the editorial office. At least twice a week the status of eligible manuscripts will be checked and if a peer reviewer has accepted an invitation to review, an email containing the S-short intervention will be generated and sent to the reviewer.

Identifying the key features of the S-short items:

The short version of the SPIRIT checklist was developed using the following steps: (i) we identified SPIRIT items with low adherence in the studies which have assessed adherence to SPIRIT (i.e. Kyte et al (18), ASPIRE UK (preliminary results, unpublished), and ASPIRE Switzerland (preliminary results, unpublished)); (ii) from these items each member of the steering committee (BS, MB, SH, AO, AWC, and Dmitry Gryaznov) selected the 10 most important items; (iii) after one round of consensus the 10 most important and poorly reported

SPIRIT items were identified. The sub-items of these 10 items which we will focus on in the intervention will be discussed amongst the steering committee. The key features as bullet points for each item will be developed in close adherence to the SPIRIT explanation and elaboration paper (14).

Control group: Usual practice:

After accepting to review an article, peer reviewers will receive the automated, standardised email with general information on how to access the manuscript, date when the peer review report is due, etc. They will not receive the additional email, sent by the investigator containing the S-short checklist.

Table 1: The ten most important and poorly reported SPIRIT items as defined by the steering group. For better understanding key features were summarised for each item (extracted from the SPIRIT explanation and elaboration paper (14)).

Item	Section	SPIRIT item	Key features
1	Outcome (12)	Primary outcome, including the specific measurement variable (eg, systolic blood pressure), analysis metric (eg, change from baseline, final value, time to event), method of aggregation (eg, median, proportion), and time point for each outcome. Explanation of the clinical relevance is strongly recommended.	• The specific measurement variable of the primary outcome which corresponds to the data collected directly from trial participants (e.g. Beck Depression Inventory score, all-cause mortality)<br>• The time point of the primary outcome (for time to event outcomes, such as overall survival, at least the follow-up period or censoring point should be reported)
2	Sample size (14)	Estimated number of participants needed to achieve study objectives and how it was determined, including clinical and statistical assumptions supporting any sample size calculations.	• Total and per group sample size (including α-value and power)<br>• Which outcome was used for the sample size calculation<br>• The values assumed for the outcome in each study group or for one study group plus effect size<br>• A rationale or reference for the outcome values assumed for each study group<br>• Type of statistical test used<br>• Adjustment for losses to follow-up, or an explanation why this was not taken into account
3	Recruitment (15)	Strategies for achieving adequate participant enrolment to reach target sample size.	• The location of recruitment (e.g. emergency department, community)<br>• The person who will identify patients (e.g. GP, surgeon, study nurse)<br>• The expected recruitment rate or duration of recruitment
4	Allocation implementation (16 c)	Who will generate the allocation sequence, who will enrol participants, and who will assign participants to interventions.	• The person(s) who will enrol/assign participants
5	Blinding (17 a)	Who will be blinded after assignment to interventions (eg, trial participants, care providers, outcome assessors, data analysts) and how.	• Blinding status of trial participants<br>• Blinding status of care providers<br>• Blinding status of outcome assessor for primary outcome
6	Data collection methods (18 a)	Plans for assessment and collection of outcome, baseline, and other trial data, including any related processes to promote data quality (eg, duplicate measurements, training of assessors) and a description of study instruments (eg, questionnaires, laboratory tests) along with their reliability and validity, if known. Reference to where data collection forms can be found, if not in the protocol.	• The person(s) who will collect data for the primary outcome
7	Data collection methods - retention (18 b)	Plans to promote participant retention and complete follow-up, including list of any outcome data to be collected for participants who discontinue or deviate from intervention protocols.	• Strategies to promote participant retention and complete follow up (e.g. phone call reminders, financial compensation)
8	Statistical methods (20 a)	Statistical methods for analysing the primary outcome. Reference to where other details of the statistical analysis plan can be found, if not in the protocol.	• The main analysis of the primary outcome including the analysis methods to be used for statistical comparisons<br>• The effect measure for the primary outcome (e.g. OR, RR, difference in means)<br>• Significance level and/or intended use of confidence intervals
9	Population analysed (20 c)	Definition of analysis population relating to protocol non-adherence (eg, as randomised analysis), and any statistical methods to handle missing data (eg, multiple imputation).	• Clear indication of which participants will be included in the main analysis. Simply stating intention-to-treat or per-protocol without further specification is not sufficient.<br>• How missing data will be handled (or a description of why missing data is unlikely)
10	Access to data (29)	Statement of who will have access to the final trial dataset, and disclosure of contractual agreements that limit such access for investigators	• A description of who will have access to the full dataset after the trial and whether individual patient data will be shared in any form with other researchers, the public and patients.

4.5 Outcomes

Primary outcome:

The primary outcome of this study will be the difference in the mean proportion of adequately reported S-short items in published articles between the two groups.

Secondary outcomes:

Secondary outcomes will include the following:

- Difference in mean proportion of adequately reported S-short items in published articles considering each sub-item (see 'Assessment of outcomes') as a separate item.
- Time (days) between the date an editor was assigned and the date of the first decision (as communicated to the author after the first round of peer review).
- Proportion of articles rejected after the first round of peer review.
- Proportion of articles published in BMJ Open.

Additional outcomes:

As peer reviewer comments are published alongside published articles on BMJ Open, we will examine peer reviewers' comments for any reference to SPIRIT and trial reporting and check whether these changes were implemented. These results might be published later as post-hoc analyses.

Data collection methods:

The outcomes will be assessed independently by two (blinded or at least partially blinded; see "blinding") outcome assessors with expertise in the design and reporting of clinical trials. Any disagreement will be resolved by consensus or if necessary by consulting a third assessor. To ensure consistency between reviewers, we will first pilot the data extraction form; any disparities in the interpretation will be discussed and the data extraction form will be modified accordingly.

Adequate reporting of items will be assessed from full-text publications using the SPIRIT checklist (14). The following included items have sub-items which will be extracted separately:

- Outcome (item 12)
    - The specific measurement variable of the primary outcome which corresponds to the data collected directly from trial participants (e.g. Beck Depression Inventory score, all-cause mortality)
    - The time point of the primary outcome (if time to event [e.g. overall survival] the follow-up period or censoring point)

- Sample size (item 14)
  - Total and per group sample size (including α-value and power)
  - Which outcome was used for sample size calculation
  - The values assumed for the outcome in each study group or for one study group plus effect size
  - A rationale or reference for the outcome values assumed for each study group
  - Type of statistical test used
  - Adjustment for losses to follow-up, or an explanation why this was not taken into account
- Recruitment (item 15)
  - The location of recruitment (e.g. emergency department, community)
  - The person who will identify patients (e.g. GP, surgeon, study nurse)
  - The expected recruitment rate or duration of recruitment
- Blinding (item 17a)
  - Blinding status of trial participants
  - Blinding status of care providers
  - Blinding status of outcome assessor for primary outcome
- Statistical methods (item 20 a)
  - The main analysis of the primary outcome including the analysis methods to be used for statistical comparisons
  - The effect measure for the primary outcome (e.g. OR, RR, difference in means)
  - Significance level and/or intended use of confidence intervals
- Population analysed (item 20 c)
  - Clear indication of which participants will be included in the main analysis. Simply stating intention-to-treat or per-protocol without further specification is not sufficient.
  - How missing data will be handled (or a description of why missing data is unlikely)

All items will be judged as either “yes” meaning adequately reported, “no” meaning not adequately reported, or “NA” meaning that this sub-item is not applicable for this protocol. Items with different sub-items will only be judged as adequately reported if all relevant sub-items are adequately reported.

Data to calculate the following outcomes will be extracted directly from ScholarOne:

- Time from assigning an academic editor until the first decision: The day when the academic editor was assigned and the day of the first decision (e.g. major revision,

minor revision, reject) will be extracted from ScholarOne to calculate the number of days until the first decision.

- Proportion of articles rejected after the first round of peer-review: Articles which were not invited for re-submission will be labelled and counted.
- Proportion of articles published: Articles which will be published in BMJ Open will be counted and collected for data extraction.

4.6 Participant timeline

The overview of the study schedule, including enrolment, intervention and assessments is presented in Table 2.

Table 2: Study schedule

	Enrolment	Allocation and intervention	Intervention	Post-intervention	
Time-point	*Studies submitted to journal*	*Eligible studies send out for peer review: After first peer reviewer accepts invitation*	*Whenever an additional peer reviewer accepts invitation*	*First decision by journal*	*Published manuscripts*
Eligibility screen	X				
Allocation		X			
Intervention:					
S-short + usual care		X	X		
Usual care		X	X		
Assessment of trial characteristics:					
Funding source					X
Study centres (single centre or multicentre)					X
Sample size					X
Study design (e.g. parallel arm, crossover)					X
Hypothesis (e.g. superiority, non-inferiority)					X
Medical field					X
Intervention tested					X
Number of trial arms					X
Number of peer-reviewers					X
Number of journals requesting SPIRIT adherence (submission of checklist mandatory)					X
Assessment of outcomes:					
Time from assigning an academic editor to first decision				X	
Proportion of articles rejected after the first round of peer review				X	
Proportion of articles published					X
Adherence to SPIRIT items and sub-items					X

4.7. Sample size

We randomly selected 20 eligible RCT protocols that were published in BMJ Open between November and December 2019 and assessed adherence to S-short. We found an overall proportional adherence of 0.53 (standard deviation (SD): 0.20).
For the sample size calculation we hypothesise that the intervention S-Short will result in a 25% relative increase in adequate reporting compared to the control (meaning that 67% of items will be adequately reported in the intervention group and 53% in the control group). To demonstrate a significant difference with a power of 90% and a type 1 error at 5% a total of 88 articles will be required in this scenario (44 per treatment arm; based on a two sided t-test). To be conservative with the sample size and to be sure that the trial is not underpowered we will increase the sample size by 20% to 106 articles (53 per treatment group). This will allow us to be also sufficiently powered when the SD is 0.22.
Since the final sample size will be based on the number of articles published, rather than on the number of manuscripts randomised, eligible RCTs will be included and randomised until the number of 53 published RCTs is reached in each arm (resulting in no less than 106 articles), to avoid loss of power due to potential imbalance between arms. After recruitment has stopped we will wait three months so that manuscripts which are still in production can be published. Manuscripts which are published after the three month period will be excluded.

4.8 Randomisation and blinding
Eligible articles for which editors decide to send out for external peer review will be randomised into one of the two groups (allocation 1:1). The randomisation list will be created by the study-randomizer system (28) using random block sizes between 2 and 8. As soon as the first peer reviewer accepts the invitation, the manuscript will be included and randomised to one of the two intervention arms. One of the investigators (BS) will log onto the study randomizer-system (28) and enter the study identification number (ID; provided by the journal system), and the study title. Subsequently, all additional peer reviewers accepting the invitation to review the same manuscript will receive the same intervention (S-short plus usual practice or usual practice only) as the first peer reviewer.

Authors will be blinded to the intervention allocation. Editors will not be informed about the randomisation (possible exception listed under “4.3 Interventions”). To avoid potential bias, peer reviewers and authors will not be informed of the study hypothesis, design and intervention. All submitting authors and invited reviewers are routinely told that BMJ has a research programme and their article/review may be included in this research.

Outcomes will be assessed in duplicate (see assessment of outcomes). At least one outcome assessor will be blinded. Due to restricted resources the investigator conducting the randomisation (BS) might be included in the data-extraction from published manuscripts.

4.9 Data management and confidentiality

Outcomes from published articles will be assessed and extracted in duplicate. Since this information is not confidential, we will use Google Forms for data extraction from published RCTs. Data entered will be validated for completeness.

Data from ScholarOne (e.g. Title of manuscript, first author, randomisation ID, date when manuscript was accepted by an editor, date when the final decision was made, final decision, number of peer reviewers who peer reviewed the manuscript, the peer review) will be extracted, anonymised and entered in a password protected database which will be stored on a server from the University of Oxford. Data will be managed and curated according to University of Oxford regulations, which includes regular back-up (on a daily basis) of the virtual drives where the data are stored.

The raw data extracted from the included manuscripts will be anonymised and made openly accessible (i.e. giving the included RCT a number instead of identifying them). Derived/aggregated data, including anonymised information generated from ScholarOne will be stored and made available to the research community when the project ends (see also "8. Publication policy and access to data"). The principal investigator (BS) and anyone else who will see the identifiable data will sign a confidentially agreement with BMJ Open, confirming that they will not share identifiable data with any other party. The BMJ Publishing Group's Privacy Statement mentions that it might share data with external researchers for quality improvement. Furthermore, peer reviewers for all BMJ journals receive the following statement in their invitation letter "*We are constantly trying to find ways of improving the peer review system and have an ongoing programme of research. If you do not wish your review entered into a study please let us know by emailing […] as soon as possible.*"

4.10 Statistical methods

4.10.1 Populations of analysis

The main population for analysis will be all manuscripts randomised and accepted for publication in BMJ Open. In contrast to RCTs conducted with patients, where drop outs need to be carefully considered (e.g. multiple imputation of missing data), we are only interested in the reporting adherence of RCTs that are published. All outcomes related to reporting completeness will be assessed on the main population for analysis. The outcomes 'proportion or articles rejected after the first round of peer review' and 'proportion of articles published' will

be assessed on the basis of all randomised manuscripts. The outcome 'time between peer review assignment and first decision' will be estimated for both the main population for analysis and all randomised manuscripts.

4.10.2 Data analysis

All quantitative variables will be described using means and standard deviations, or median and interquartile ranges in case severe departures from a normal distribution are identified. Data distribution will be inspected visually (i.e. by histograms) instead of performing formal statistical tests for normality. Categorical variables will be described using frequencies and percentages. For the primary and secondary outcomes, we will estimate the difference between means between the two groups and report them with respective 95% confidence intervals.

4.10.3 Analysis of primary endpoint

The primary outcome will be the difference in the mean proportion of adequately reported S-short items in published articles between the two groups. If the data on the primary outcome is normally distributed then the two groups (i.e. S-short plus usual practice vs. usual practice) will be compared using an unpaired Student's t-test to compare the unadjusted mean proportion of adequate reporting. If the data is not normally distributed, comparisons will be performed using a non-parametric equivalent test (i.e. Wilcoxon-Mann-Whitney test for testing whether the population medians of the two groups are the same).

For the analyses of the primary outcomes a p-value of 0.05 (5% significance level) will be used to indicate statistical significance and treatment effect (mean difference) reported with 95% confidence intervals (or median and respective interquartile ranges, in case of asymmetric distribution). Exact p-values will be presented up to three decimal places. We anticipate there will be no missing data in this study, neither at the individual S-short items, nor at the manuscript level (i.e. ScholarOne). This is due to the study design, which will include only the randomised manuscripts that are accepted for publication.

4.10.4 Analysis of secondary endpoints

To investigate the effect of the intervention on the secondary outcomes, mean differences with respective 95% confidence intervals will also be reported for these outcomes. If normality is not observed for any of the continuous secondary outcomes, the same strategy adopted for the primary outcome (use of a non-parametric equivalent to the Student's *t*-test) will be used.

A p-value of 0.05 will indicate statistical significance for the observed treatment effect on the secondary outcomes. Exact p-values will be presented up to three decimal places. Similar to

the primary outcome, we anticipate there will be no missing data for any of the secondary outcomes, as we will have access to ScholarOne where all relevant information is automatically collected.

4.10.5 Pre-specified subgroup analysis

No formal subgroup comparative analysis is planned for the primary or secondary outcomes. However, the effect of the intervention on the primary outcome within subgroups, will be presented using forest plots to visually examine whether it differs according to the sample size ($n < 100$ vs. $n \geq 100$) reported in the included protocols, as there is evidence that larger sample size is associated with higher adherence to reporting guidelines (8). This analysis will be exploratory, with the aim of supporting new hypothesis generation, rather than conclusive.

5 Legal and general logistics

5.1. Organisation of study

5.1.1 Coordinating centre

The coordinating centre will be the Centre for Statistics in Medicine at the University of Oxford under the responsibilities of Prof Sally Hopewell and Dr Benjamin Speich.

The coordinating centre will ensure the following missions:

- Training of the staff
- Implementation of quality control
- Logical controls of data
- Follow-up on requests for correction/validation
- Statistical analysis
- Data curation

5.1.2 Scientific committee

The scientific committee is composed of:

- Prof Sally Hopewell, Centre for Statistics in Medicine, Oxford University, UK
- Dr Sara Schroter, The BMJ, London, UK
- Prof Matthias Briel, University of Basel, Switzerland
- Dr Ayodele Odutayo, St Michael's Hospital, Toronto, Canada
- Prof An-Wen Chan, University of Toronto, Canada
- Dr Benjamin Speich, Centre for Statistics in Medicine, University of Oxford, UK
- Dr Michael M Schlussel, UK EQUATOR Centre, Centre for Statistics in Medicine, University of Oxford, UK

The scientific committee is in charge of:

- Participating in the elaboration of the protocol
- Defining and validating the key features for each SPIRIT item
- Following the evolution of the committed study
- Publishing the results of this study

5.2. Regulatory aspects

Ethical approval for this study will be sought from the Central University Research Ethics Committee (CUREC) of the University of Oxford. Any amendments in the conduct of the study, collection of outcomes or analysis will be reported to the CUREC. The tested intervention has the goal to improve the quality of published articles (i.e. the adherence to SPIRIT) and could also be implemented as usual practice without testing at the journal level. In agreement with two other studies, testing similar interventions (26, 29), we think that it is ethical to conduct this study without obtaining written consent. The main reasons for this decision are the following:

- Informing the authors and peer reviewers would make it impossible to measure the effect of our intervention. By informing peer reviewers and authors we would create an artificial context which is not comparable to the "real world context". Authors and peer reviewers would most likely be much more aware of SPIRIT if they received information about the study. Furthermore, simply knowing you are participating in a study could strongly influence the natural behaviour of peer reviewers and authors (e.g. putting more effort into reviewing or revising a manuscript than under "real world conditions").
- The intervention does not pose any risk of harms for authors or peer reviewers.
- The intervention is not a medical intervention but is aimed at improving the research quality and journal processes.
- BMJ Publishing Group has a Privacy Statement in place which clearly mentions that research programmes might be in place for quality improvement.
- The intervention could be part of routine practice at any journal without previous assessment of its efficacy.
- No data which identifies participating manuscripts will be published.

6 Publication policy and access to data

The results from this study will be published in a peer reviewed journal irrespective of the study results. Authorship for publications will be granted according to the rules of the International Committee of Medical Journal Editors (ICMJE). We plan to publish the full anonymised dataset as a supplementary file together with the main publication.

References

1. Altman DG, Simera I, Hoey J, Moher D, Schulz K. EQUATOR: reporting guidelines for health research. Lancet. 2008;371(9619):1149-50.
2. Simera I. EQUATOR Network collates resources for good research. BMJ. 2008;337:a2471.
3. Simera I, Altman DG. Writing a research article that is "fit for purpose": EQUATOR Network and reporting guidelines. Evid Based Med. 2009;14(5):132-4.
4. Simera I, Altman DG, Moher D, Schulz KF, Hoey J. Guidelines for reporting health research: the EQUATOR network's survey of guideline authors. PLoS Med. 2008;5(6):e139.
5. Simera I, Moher D, Hirst A, Hoey J, Schulz KF, Altman DG. Transparent and accurate reporting increases reliability, utility, and impact of your research: reporting guidelines and the EQUATOR Network. BMC Med. 2010;8:24.
6. Simera I, Moher D, Hoey J, Schulz KF, Altman DG. The EQUATOR Network and reporting guidelines: Helping to achieve high standards in reporting health research studies. Maturitas. 2009;63(1):4-6.
7. Enhancing the QUAlity and Transparancy Of health Research. The EQUATOR netowrk. https://www.equator-network.org/about-us/what-is-a-reporting-guideline/ [acessed: 18 MArch 2020].
8. Jin Y, Sanger N, Shams I, Luo C, Shahid H, Li G, et al. Does the medical literature remain inadequately described despite having reporting guidelines for 21 years? - A systematic review of reviews: an update. Journal of multidisciplinary healthcare. 2018;11:495-510.
9. Duley L, Antman K, Arena J, Avezum A, Blumenthal M, Bosch J, et al. Specific barriers to the conduct of randomized trials. Clin Trials. 2008;5(1):40-8.
10. Collins R, MacMahon S. Reliable assessment of the effects of treatment on mortality and major morbidity, I: clinical trials. Lancet. 2001;357(9253):373-80.
11. Chalmers I. Unbiased, relevant, and reliable assessments in health care: important progress during the past century, but plenty of scope for doing better. BMJ. 1998;317(7167):1167-8.
12. Kasenda B, von Elm E, You J, Blumle A, Tomonaga Y, Saccilotto R, et al. Prevalence, characteristics, and publication of discontinued randomized trials. JAMA. 2014;311(10):1045-51.
13. Chan AW, Tetzlaff JM, Altman DG, Laupacis A, Gotzsche PC, Krleza-Jeric K, et al. SPIRIT 2013 statement: defining standard protocol items for clinical trials. Ann Intern Med. 2013;158(3):200-7.
14. Chan AW, Tetzlaff JM, Gotzsche PC, Altman DG, Mann H, Berlin JA, et al. SPIRIT 2013 explanation and elaboration: guidance for protocols of clinical trials. BMJ. 2013;346:e7586.
15. Chan AW, Hrobjartsson A. Promoting public access to clinical trial protocols: challenges and recommendations. Trials. 2018;19(1):116.
16. Li T, Boutron I, Al-Shahi Salman R, Cobo E, Flemyng E, Grimshaw JM, et al. Review and publication of protocol submissions to Trials - what have we learned in 10 years? Trials. 2016;18(1):34.
17. Spence O, Hong K, Onwuchekwa Uba R, Doshi P. Availability of study protocols for randomized trials published in high-impact medical journals: A cross-sectional analysis. Clin Trials. 2020;17(1):99-105.
18. Kyte D, Duffy H, Fletcher B, Gheorghe A, Mercieca-Bebber R, King M, et al. Systematic evaluation of the patient-reported outcome (PRO) content of clinical trial protocols. PLoS One. 2014;9(10):e110229.
19. Shamseer L, Hopewell S, Altman DG, Moher D, Schulz KF. Update on the endorsement of CONSORT by high impact factor journals: a survey of journal "Instructions to Authors" in 2014. Trials. 2016;17(1):301.

20. Hopewell S, Altman DG, Moher D, Schulz KF. Endorsement of the CONSORT Statement by high impact factor medical journals: a survey of journal editors and journal 'Instructions to Authors'. Trials. 2008;9:20.
21. Hopewell S, Ravaud P, Baron G, Boutron I. Effect of editors' implementation of CONSORT guidelines on the reporting of abstracts in high impact medical journals: interrupted time series analysis. BMJ. 2012;344:e4178.
22. Blanco D, Kirkham JJ, Altman DG, Moher D, Boutron I, Cobo E. Interventions to improve adherence to reporting guidelines in health research: a scoping review protocol. BMJ Open. 2017;7(11):e017551.
23. Blanco D, Altman D, Moher D, Boutron I, Kirkham JJ, Cobo E. Scoping review on interventions to improve adherence to reporting guidelines in health research. BMJ Open. 2019;9(5):e026589.
24. Hopewell S, Collins GS, Boutron I, Yu LM, Cook J, Shanyinde M, et al. Impact of peer review on reports of randomised trials published in open peer review journals: retrospective before and after study. BMJ. 2014;349:g4145.
25. Cobo E, Selva-O'Callagham A, Ribera JM, Cardellach F, Dominguez R, Vilardell M. Statistical reviewers improve reporting in biomedical articles: a randomized trial. PLoS One. 2007;2(3):e332.
26. Speich B, Schroter S, Briel M, Moher D, Puebla I, Clark A, et al. Impact of a short version of the CONSORT checklist for peer reviewers to improve the reporting of randomised controlled trials published in biomedical journals: study protocol for a randomised controlled trial. BMJ Open. 2020;10(3):e035114.
27. Chauvin A, Ravaud P, Baron G, Barnes C, Boutron I. The most important tasks for peer reviewers evaluating a randomized controlled trial are not congruent with the tasks most often requested by journal editors. BMC Med. 2015;13:158.
28. Study Randomizer. https://studyrandomizer.com/ [accessed: 8. July 2019].
29. Hair K, Macleod MR, Sena ES. A randomised controlled trial of an Intervention to Improve Compliance with the ARRIVE guidelines (IICARus). bioRxiv. 2018.

Appendix

Appendix 1: Example of the email which will be sent to reviewers in the intervention arm (S-Short). The exact wording might be slightly adapted according to the editor's preference.

Dear **Title, Name**,

Thank you for agreeing to peer review a manuscript for BMJ Open. We are trying to improve the reporting of protocols for randomised controlled trials according to the SPIRIT guidelines and would like you to check whether the following most important and poorly reported items are adequately addressed as indicated in the attached table or alternatively listed below.

Item	Section	SPIRIT item	Key features
1	Outcome (12)	Primary outcome, including the specific measurement variable (eg, systolic blood pressure), analysis metric (eg, change from baseline, final value, time to event), method of aggregation (eg, median, proportion), and time point for each outcome. Explanation of the clinical relevance is strongly recommended.	• The specific measurement variable of the primary outcome which corresponds to the data collected directly from trial participants (e.g. Beck Depression Inventory score, all-cause mortality)<br>• The time point of the primary outcome (for time to event outcomes, such as overall survival, at least the follow-up period or censoring point should be reported)
2	Sample size (14)	Estimated number of participants needed to achieve study objectives and how it was determined, including clinical and statistical assumptions supporting any sample size calculations.	• Total and per group sample size (including α-value and power)<br>• Which outcome was used for the sample size calculation<br>• The values assumed for the outcome in each study group or for one study group plus effect size<br>• A rationale or reference for the outcome values assumed for each study group<br>• Type of statistical test used<br>• Adjustment for losses to follow up, or an explanation why this was not taken into account
3	Recruitment (15)	Strategies for achieving adequate participant enrolment to reach target sample size	• The location of recruitment (e.g. emergency department, community)<br>• The person who will identify patients (e.g. GP, surgeon, study nurse)<br>• The expected recruitment rate or duration of recruitment
4	Allocation implementation (16 c)	Who will generate the allocation sequence, who will enrol participants, and who will assign participants to interventions.	• The person(s) who will enrol/assign participants
5	Blinding (17 a)	Who will be blinded after assignment to interventions (eg, trial participants, care providers, outcome assessors, data analysts) and how.	• Blinding status of trial participants<br>• Blinding status of care providers<br>• Blinding status of outcome assessor for primary outcome
6	Data collection methods (18 a)	Plans for assessment and collection of outcome, baseline, and other trial data, including any related processes to promote data quality (eg, duplicate measurements, training of assessors) and a description of study instruments (eg, questionnaires, laboratory tests) along with their reliability and validity, if known. Reference to where data collection forms can be found, if not in the protocol	• The person(s) who will collect data for the primary outcome
7	Data collection methods - retention (18 b)	Plans to promote participant retention and complete follow-up, including list of any outcome data to be collected for participants who discontinue or deviate from intervention protocols.	• Strategies to promote participant retention and complete follow up (e.g. phone call reminders, financial compensation)
8	Statistical methods (20 a)	Statistical methods for analysing the primary outcome. Reference to where other details of the statistical analysis plan can be found, if not in the protocol.	• The main analysis of the primary outcome including the analysis methods to be used for statistical comparisons<br>• The effect measure for the primary outcome (e.g. OR, RR, difference in means)<br>• Significance level and/or intended use of confidence intervals
9	Population analysed (20 c)	Definition of analysis population relating to protocol non-adherence (eg, as randomised analysis), and any statistical methods to handle missing data (eg, multiple imputation).	• Clear indication of which participants will be included in the main analysis. Simply stating intention-to-treat or per-protocol without further specification is not sufficient.<br>• How missing data will be handled (or a description of why missing data is unlikely)
10	Access to data (29)	Statement of who will have access to the final trial dataset, and disclosure of contractual agreements that limit such access for investigators	• A description of who will have access to the full dataset after the trial and whether individual patient data will be shared in any form with other researchers, the public and patients.

Your efforts are highly appreciated.

Best wishes,

Editorial Production Assistant

BMJ Open, Editorial Office

Please ensure as a minimum that each of the following elements are clearly described:

Outcome (Item 12)

- The specific measurement variable of the primary outcome which corresponds to the data collected directly from trial participants (e.g. Beck Depression Inventory score, all-cause mortality)
- The time point of the primary outcome (for time to event outcomes, such as overall survival, at least the follow-up period or censoring point should be reported)

Sample size (Item 14)

- Total and per group sample size (including α-value and power)
- Which outcome was used for the sample size calculation
- The values assumed for the outcome in each study group or for one study group plus effect size
- A rationale or reference for the outcome values assumed for each study group
- Type of statistical test used
- Adjustment for losses to follow-up, or an explanation why this was not taken into account

Recruitment (Item 15)

- The location of recruitment (e.g. emergency department, community)
- The person who will identify patients (e.g. GP, surgeon, study nurse)
- The expected recruitment rate or duration of recruitment

Allocation implementation (Item 16 c)

- The person(s) who will enrol/assign participants

Blinding (Item 17a)

- Blinding status of trial participants
- Blinding status of care providers
- Blinding status of outcome assessor for primary outcome

Data collection methods (Item 18 a)

- The person(s) who will collect data for the primary outcome

Data collection methods - retention (Item 18 b)

- Strategies to promote participant retention and complete follow up (e.g. phone call reminders, financial compensation)

Statistical methods (Item 20 a)

- The main analysis of the primary outcome including the analysis methods to be used for statistical comparisons
- The effect measure for the primary outcome (e.g. OR, RR, difference in means)
- Significance level and/or intended use of confidence intervals

Population analysed (Item 20 c)

- Clear indication of which participants will be included in the main analysis. Simply stating intention-to-treat or per-protocol without further specification is not sufficient.
- How missing data will be handled (or a description of why missing data is unlikely)

Access to data (Item 29)

A description of who will have access to the full dataset after the trial and whether individual patient data will be shared in any form with other researchers, the public and patients.
