# Supplementary material for: Reminding Peer Reviewers of Reporting Guideline Items to Improve Completeness in Published Articles: Primary Results of 2 Randomized Trials
Source: JAMA Netw Open. 2023 Jun 9;6(6):e2317651. doi: 10.1001/jamanetworkopen.2023.17651 (PMC10257091; doi:10.1001/jamanetworkopen.2023.17651)
Supplement: Supplement 3. — eAppendix 1. Eligibility Criteria for CONSORT-PR and SPIRIT-PR eAppendix 2. Example of the Intervention Email Sent in the CONSORT-PR Study for BMJ Open eAppendix 3. Selection of the 10 Most Important and Poorly Reported Reporting Items and Development of the Brief Explanation for Each Item eAppendix 4. Assumptions for Sample Size Calculations eAppendix 5. Stata Code for the Primary Outcome eTable 1. The Ten Most Important and Underreported SPIRIT Items as Defined by a Group of Experts eTable 2. Proportion of Manuscripts Included in the Analysis of the CONSORT-PR Trial Per Participating Journal eTable 3. Medical Specialties of Included Manuscripts eTable 4. Sensitivity Analysis of the Primary Outcome for the CONSORT-PR Trial eTable 5. The Difference in the Mean Proportion of Adequate Reporting of the 10 Selected Reporting Items, Stratified by Sample Size and Journal Impact Factor eFigure. Histograms eReferences [file jamanetwopen-e2317651-s003.pdf]

Supplementary Online Content

Speich B, Mann E, Schönenberger CM, et al. Reminding peer reviewers of reporting guideline items to improve completeness in published articles: primary results of 2 randomized trials. *JAMA Netw Open*. 2023;6(6):e2317651. doi:10.1001/jamanetworkopen.2023.17651

eAppendix 1. Eligibility Criteria for CONSORT-PR and SPIRIT-PR

eAppendix 2. Example of the Intervention Email Sent in the CONSORT-PR Study for BMJ Open

eAppendix 3. Selection of the 10 Most Important and Poorly Reported Reporting Items and Development of the Brief Explanation for Each Item

eAppendix 4. Assumptions for Sample Size Calculations

eAppendix 5. Stata Code for the Primary Outcome

eTable 1. The Ten Most Important and Underreported SPIRIT Items as Defined by a Group of Experts

eTable 2. Proportion of Manuscripts Included in the Analysis of the CONSORT-PR Trial Per Participating Journal

eTable 3. Medical Specialties of Included Manuscripts

eTable 4. Sensitivity Analysis of the Primary Outcome for the CONSORT-PR Trial

eTable 5. The Difference in the Mean Proportion of Adequate Reporting of the 10 Selected Reporting Items, Stratified by Sample Size and Journal Impact Factor

eFigure. Histograms

eReferences

This supplementary material has been provided by the authors to give readers additional information about their work.

© 2023 Speich B et al. *JAMA Network Open.*

eAppendix 1. Eligibility Criteria for CONSORT-PR and SPIRIT-PR

<u>CONSORT-PR:</u>

Inclusion criteria for journals:

Journals were eligible for participation if they

(i) endorsed the CONSORT statement by mentioning it in the journals' Instruction to authors
(ii) had published primary results of at least five RCTs in 2017 (identified using a PubMed search).

Inclusion criteria for manuscripts:

All new manuscript submissions that reported the primary results of RCTs, which the journal editor had decided to send out for external peer review.

Exclusion criterial for manuscripts:

We excluded

(i) Manuscripts clearly presenting secondary trial results, additional time points, economic analyses or other analyses
(ii) Manuscripts that were clearly labelled as a pilot, feasibility or animal studies
(iii) Manuscripts not sent for peer review.

<u>SPIRIT-PR:</u>

Inclusion criteria for journals:

It was planned from the beginning that this study will only be conducted at the BMJ Open as it publishes numerous protocols. Hence, no inclusion criteria for the journal were specified.

Inclusion criteria for manuscripts:

All submitted manuscripts sent out for external review that described protocols for RCTs.

Exclusion criterial for manuscripts:

We excluded

(i) Manuscripts which were clearly labelled as a pilot or feasibility study
(ii) Studies randomizing animals or cells
(iii) Separate publications of data analysis plans

© 2023 Speich B et al. *JAMA Network Open.*

eAppendix 2. Example of the Intervention Email Sent in the CONSORT-PR Study for BMJ Open

Sent from: BMJOpen@bmj.com

Email Subject: Peer review for BMJ Open: appeal to consider CONSORT items

Dear **Title, Name**,

Thank you for agreeing to peer review a manuscript for BMJ Open. We are trying to improve the reporting of randomized controlled trials according to the CONSORT guidelines, and would like you to check whether the following most important and poorly reported items are adequately addressed as indicated in the attached table or alternatively listed below.

If you have the impression that some of the items are not adequately reported, please include this in your review report and do not reply directly to this email.

Your efforts are highly appreciated.

Sincerely,

BMJ Open Editorial Office

1) <u>Outcomes (Item 6a)</u>

Completely defined pre-specified primary outcome measure including how and when it was assessed

Is it clear (1) what the primary outcome is (usually the one used in the sample size calculation), (2) how it was measured (if relevant; e.g. which score used), (3) at what time point, and (4) what the analysis metric was (e.g. change from baseline, final value)?

2) <u>Sample size (Item 7a)</u>

How sample size was determined

Is there a clear description of how the sample size was determined, including (1) the estimated outcomes in each group; (2) the α (type I) error level; (3) the statistical power (or the β (type II) error level); and (4) for continuous outcomes, the standard deviation of the measurements?

3) <u>Sequence generation (Item 8a)</u>

Method used to generate random allocation sequence

Does the description make it clear if the "assigned intervention is determined by a chance process and cannot be predicted"?

4) <u>Allocation concealment (Item 9)</u>

© 2023 Speich B et al. *JAMA Network Open.*

Mechanism used to implement random allocation sequence (such as sequentially numbered containers), describing any steps taken to conceal the sequence until interventions were assigned
Is it clear how the care provider enrolling participants was made ignorant of the next assignment in the sequence (different from blinding)? Possible methods can rely on centralized or "third-party" assignment (i.e., use of a central telephone randomization system, automated assignment system, sealed containers).

5) Blinding (Item 11a)
If done, who was blinded after assignment to interventions (for example, participants, care providers, those assessing outcomes)
Is it clear if (1) healthcare providers, (2) patients, and (3) outcome assessors are blinded to the intervention? General terms such as "double-blind" without further specifications should be avoided.

6) Outcomes and estimation (Item 17a/b)
For the primary outcome, results for each group, and the estimated effect size and its precision (such as 95% confidence intervals)
Is the estimated effect size and its precision (such as standard deviation or 95% confidence intervals) for each treatment arm reported? When the primary outcome is binary, both the relative effect (risk ratio, relative risk) or odds ratio) and the absolute effect (risk difference) should be reported with confidence intervals.

7) Harms (Items 19)
All important harms or unintended effects in each group
Is the number of affected persons in each group, the severity grade (if relevant) and the absolute risk (e.g. frequency of incidence) reported? Are the number of serious, life threatening events and deaths reported? If no adverse event occurred this should be clearly stated.

8) Registration (Item 23)
Registration number and name of trial registry
Is the registry and the registration number reported? If the trial was not registered, it should be explained why.

9) Protocol (Item 24)
Where trial protocol can be accessed
Is it stated where the trial protocol can be assessed (e.g. published, supplementary file, repository, directly from author, confidential and therefore not available)?

10) Funding (Item 25)
Sources of funding and other support (such as supply of drugs) and role of funders
Are (1) the funding sources, and (2) the role of the funder(s) described?

© 2023 Speich B et al. *JAMA Network Open.*

eAppendix 3. Selection of the 10 Most Important and Poorly Reported Reporting Items and Development of the Brief Explanation for Each Item

CONSORT-PR:

As described in the study protocol (1), the items were selected using the following approach "*For the development of C-short we chose the 10 most important and poorly reported CONSORT items as identified by a group of CONSORT experts in a previous study conducted by Hopewell and colleagues (2). The selection of the items was based on expert opinion and empirical evidence whenever available (2).*"

The short explanation for each item was "*extracted and amended from the CONSORT explanation and elaboration paper (3) and from COBWEB which is an online writing aid tool (2). The short explanation was discussed and adapted by the scientific committee*."(1)

SPIRIT-PR

The short version of the SPIRIT checklist was developed using the following steps: (i) we identified SPIRIT items with low adherence in the studies which have assessed adherence to SPIRIT (i.e. Kyte et al (4), ASPIRE-UK (5), and ASPIRE Switzerland, Canada and Germany (ASPIRE-SCAGE (6)); (ii) from these items each member of the steering committee (BS, MB, SH, AO, and AWC) as well, and Dmitry Gryaznov (first author of ASPIRE-SCAGE (6)) selected the 10 most important items; (iii) after one round of consensus the 10 most important and poorly reported SPIRIT items were identified. The sub-items of these 10 items which we focused on in the intervention were discussed amongst the steering committee. The key features as bullet points for each item were developed in close adherence to the SPIRIT explanation and elaboration paper (7).

© 2023 Speich B et al. *JAMA Network Open.*

eAppendix 4. Assumptions for Sample Size Calculations

CONSORT-PR:

We hypothesized that the mean adherence in the control group would be 71% (based on recent evidence (2) and considering that the items “Registration”, “Protocol”, and “Funding” are thoroughly checked in PLOS journals given their policy requirement; see protocol for more details (1)). Using a two-sided t-test, a sample size of 166 (83 per treatment arm) had a power of 80% to find a 15% relative increase (71% adherence in control group; 82% adherence in intervention group; standard deviation [SD] =0.25; a type 1 error at 5%). Recruitment was stopped when it was clear that sufficient manuscripts were enrolled to reach the 83 published articles (i.e. not randomized articles) per treatment arm.

SPIRIT-PR

Between November and December 2019 we selected randomly 20 eligible published RCT protocols that were published in BMJ Open. The mean adherence to the SPIRIT reporting guideline for the 10 selected most important and poorly reported SPIRIT items was assessed to be 53% (SD 0.20). Using a two-sided t-test (type I error 5%; power 90%) a sample size of 88 was required to show a relative increase of 25% (intervention: mean adherence of 67% vs 53% in control group). To be conservative and allow for a higher variance of the data (SD 0.22) we increased the sample size to 106 (53 per treatment arm). Recruitment was stopped when it was clear that sufficient manuscripts were enrolled to reach the 53 published articles (i.e. not randomized articles) per treatment arm.

© 2023 Speich B et al. *JAMA Network Open.*

eAppendix 5. Stata Code for the Primary Outcome

<u>CONSORT-PR:</u>

- gen outcome=0
- replace outcome=1 if Outcome1defineprimaryoutco+ Outcome2Howitwasmeasured+ Outcome3Thetimepointof+ Outcome4whattheanalysisme==4
- gen samplesize=0
- replace samplesize=1 if Samplesize1theestimatedou+ Samplesize2theαtypeIe+ Samplesize3thestatistical+ Samplesize4forcontinuous==4
- gen blinding=0
- replace blinding=1 if Blinding1Blindingstatusof+ Blinding2Blindingstatusof+ Blinding3Blindingstatusof==3
- gen funding=0
- replace funding=1 if Funding1thefundingsources+ Funding2theroleofthefund==2
-
- gen reportingprimary=.
- replace reportingprimary= outcome+ samplesize+ SequencegenerationDoesthede+AllocationconcealmentIsitcl+ blinding+ OutcomesandestimationIsthe+ HarmsIsthenumberofaffected+ RegistrationIstheregistryan + ProtocolisitstatedwheretheI+ funding
- ttest reportingprimary, by(Randomisationarm)

<u>SPIRIT-PR</u>

- gen outcome=0
- replace outcome=1 if Outcome1Thespecificmeasur+ Outcome2Thetimepointoft==2
- gen samplesize=0
- replace samplesize=1 if Samplesize1Totalandperg+ Samplesize2Whichoutcomew+ Samplesize3Thevaluesas+ Samplesize4Arationaleor+ Samplesize5Typeofstatist+ Samplesize6Adjustmentfor==6
- gen recruitment=0
- replace recruitment=1 if Recruitment1Thelocationof+ Recruitment2Thepersonwho+ Recruitment3Theexpectedre==3
- gen blinding=0
- replace blinding=1 if Blinding1Blindingstatusof+ Blinding2Blindingstatusof+ Blinding3Blindingstatusof==3
- gen statistics=0
- replace statistics=1 if Statisticalmethods1Themai+ Statisticalmethods2Theeff+ Statisticalmethods3Signifi==3
- gen population=0
- replace population=1 if Populationanalysis1Cleari+ Populationanalysis2Howmis==2
- gen reportingprimary=.
- replace reportingprimary= outcome+ samplesize+ recruitment+ blinding+ statistics+ population+ AllocationimplementationThe+ DatacollectionmethodsTheper+ Datacollectionmethodsretenti+ AccesstodataAdescription
- ttest reportingprimary, by(Randomisationarm)

© 2023 Speich B et al. *JAMA Network Open.*

eTable 1: The Ten Most Important and Underreported SPIRIT Items as Defined by a Group of Experts. For better understanding key features were summarized (extracted from the SPIRIT explanation and elaboration paper (7)

Item	Section	SPIRIT item	Key features
1	Outcomes (12)	Primary outcome, including the specific measurement variable (eg, systolic blood pressure), analysis metric (eg, change from baseline, final value, time to event), method of aggregation (eg, median, proportion), and time point for each outcome. Explanation of the clinical relevance is strongly recommended.	• The specific measurement variable, which corresponds to the data collected directly from trial participants (e.g. Beck Depression Inventory score, all-cause mortality)<br>• Time point (if time to event [e.g. overall survival] the follow-up period or censoring point should be described)
2	Sample size (14)	Estimated number of participants needed to achieve study objectives and how it was determined, including clinical and statistical assumptions supporting any sample size calculations.	• Total and per group sample size should be clear (including α-value and power)<br>• Outcome used for sample size calculation should be mentioned<br>• The values assumed for the outcome in each study group or for one study group plus effect size<br>• A rationale or reference for the outcome values assumed for each study group<br>• Type of statistical test provided<br>• Adjustment for losses to follow-up, if relevant, described
3	Recruitment (15)	Strategies for achieving adequate participant enrolment to reach target sample size.	• Location of recruitment described (e.g. emergency department, community)<br>• Person identifying patients described (e.g. GP, surgeon, study nurse)<br>• Expected recruitment rate or duration or recruitment stated
4	Allocation implementation (16 c)	Who will generate the allocation sequence, who will enrol participants, and who will assign participants to interventions.	• Person who will enrol/assign participants described
5	Blinding (17 a)	Who will be blinded after assignment to interventions (eg, trial participants, care providers, outcome assessors, data analysts) and how.	• Blinding status of trial participants clear<br>• Blinding status of care providers clear<br>• Blinding status of outcome assessor for primary outcome clear
6	Data collection methods (18 a)	Plans for assessment and collection of outcome, baseline, and other trial data, including any related processes to promote data quality (eg, duplicate measurements, training of assessors) and a description of study instruments (eg, questionnaires, laboratory tests) along with their reliability and validity, if known. Reference to where data collection forms can be found, if not in the protocol.	• Personnel who will collect data for the primary outcome
7	Data collection methods - retention (18 b)	Plans to promote participant retention and complete follow-up, including list of any outcome data to be collected for participants who discontinue or deviate from intervention protocols.	• Strategies to promote participant retention and complete follow up (e.g. phone call reminders, financial compensation)
8	Statistical methods (20 a)	Statistical methods for analysing the primary outcome. Reference to where other details of the statistical analysis plan can be found, if not in the protocol.	• The main analysis of the primary outcome, including the analysis methods to be used for statistical comparisons<br>• Effect measure for primary outcome specified (e.g. OR, RR, difference in means)<br>• Significance level and/or intended use of confidence intervals (at least one of this two) specified
9	Population analysed (20 c)	Definition of analysis population relating to protocol non-adherence (eg, as randomised analysis), and any statistical methods to handle missing data (eg, multiple imputation).	• Which participants will be included in the main analysis? Simply stating intention-to-treat or per-protocol without further specification is not sufficient.<br>• How missing data will be handled (or description why missing data unlikely)
10	Access to data (29)	Statement of who will have access to the final trial dataset, and disclosure of contractual agreements that limit such access for investigators	• Who will have access to full dataset after the trial? Is it intended to share individual patient data in any form with other researchers, public and patients?

© 2023 Speich B et al. *JAMA Network Open.*

eTable 2: Proportion of Manuscripts Included in the Analysis of the CONSORT-PR Trial Per Participating Journal

Journal	Number Screened	Number Randomized	% of manuscripts included in analyses that were randomized
**The BMJ**	9984	119	36% (43/119)
**BMJ Open**	14275	99	56% (55/99)
**BJSM**	1763	27	44% (12/27)
**BJO**	4152	35	60% (21/35)
**Heart**	2781	17	47% (8/17)
**PLOS One**	1044	171	48% (82/171)
**PLOS Medicine**	68	42	52% (22/42)

Abbreviations: BJSM= British Journal of Sports Medicine; BJO=British Journal of Ophthalmology

© 2023 Speich B et al. *JAMA Network Open.*

eTable 3: Medical Specialties of Included Manuscripts

Medical specialty	CONSORT-PR			SPIRIT-PR		
	Intervention group: Reminder to peer reviewers (n=122)	Control group (n=121)	Total (n=243)	Intervention group: Reminder to peer reviewers (n= 90)	Control group (n=88)	Total (n=178)
Ophthalmology	12 (9.9%)	11 (9.1%)	23 (9.5%)	1 (1.1%)	1 (1.1%)	2 (1.1%)
Cardiovascular	11 (9.1%)	11 (9.1%)	22 (9.1%)	11 (12.2%)	9 (10.2%)	20 (11.2%)
Orthopaedics	13 (10.7%)	6 (5.0%)	19 (7.9%)	3 (3.3%)	2 (2.3%)	5 (2.8%)
Paediatric	13 (10.7%)	6 (5.0%)	19 (7.8%)	6 (6.7%)	13 (14.8%)	19 (10.7%)
Public health	6 (4.9%)	10 (8.3%)	16 (6.6%)	3 (3.3%)	2 (2.3%)	5 (2.8%)
Gynaecology/Maternity/Obstetrics	7 (5.7%)	8 (6.6%)	15 (6.2%)	7 (7.8%)	0 (0%)	7 (3.9%)
Psychiatry/Psychology	8 (6.6%)	5 (4.1%)	13 (5.3%)	4 (4.4%)	3 (3.4%)	7 (3.9%)
Oncology	3 (2.5%)	5 (4.1%)	8 (3.3%)	6 (6.7%)	7 (8.0%)	13 (7.3%)
Neurology	1 (0.8%)	3 (2.5%)	4 (1.7%)	4 (4.4%)	9 (10.2%)	13 (7.3%)
Intensive care	0 (0%)	1 (0.8%)	1 (0.4%)	4 (4.4%)	7 (8.0%)	11 (6.2%)
Surgery	2 (1.7%)	2 (1.7%)	4 (1.7%)	7 (7.8%)	7 (8.0%)	14 (7.9%)
Other[a]	46 (37.7%)	53 (43.8%)	99 (40.7%)	34 (37.8%)	28 (31.8%)	62 (34.8%)

[a]CONSORT-PR: Anesthesia (n=14), infectious diseases (n=13), COVID-19 (n=10), endocrinology (n=6), gastrointestinal (n=6), respiratory (n=5), trial methodology/meta-research (n=5), dermatology (n=4), nephrology (n=4), rheumatology (n=4), physical therapy (n=4), polypharmacy (n=4), dentistry (n=3), primary care (n=3), ear-nose-throat (n=2), sport medicine (n=2), urology (n=2), cardiothoracic (n=1), emergency medicine (n=1), hepatology (n=1), obesity (n=1), reproductive medicine (n=1), alternative medicine (n=1), geriatrics (n=1), internal medicine (n=1); SPIRIT-PR: Endocrinology (n=7), respiratory (n=7), infectious diseases (n=6), physical therapy (n=5), COVID-19 (n=4), Gastrointestinal (n=4), Rheumatology (n=4), Dermatology (n=3), Hematology (n=3), Dentistry (n=3), ear-nose-throat (n=2), Nephrology (n=2), emergency medicine (n=2), women`s empowerment (n=2), anesthesia (n=1), cardiothoracic (n=1), alternative medicine (n=1), hepatology (n=1), immune mediated inflammatory disease (n=1), nursing (n=1), primary care (n=1), geriatrics (n=1).

Abbreviations: Abbreviations: CONSORT=CONsolidated Standards for Reporting Trials; PR=Peer reviewer

© 2023 Speich B et al. *JAMA Network Open.*

eTable 4: Sensitivity Analysis of the Primary Outcome for the CONSORT-PR Trial: the difference in the mean proportion of adequate reporting of the 10 selected CONSORT items after excluding manuscripts that did not present primary results for a randomized trial (n=4) or for which the intervention could not be sent out before reviewers' reports were submitted (n=1).

	**Intervention group: Reminder to peer reviewers**	**Control group**	**Mean difference**	**p-value**
**CONSORT-PR trial:**	n=120	n=118		
Proportion of adequately reported CONSORT items	69.2%<br>(65.7-72.6% 95% CI)	67.0%<br>(62.9-71.2% 95% CI)	2.1%<br>(-3.2-7.5% 95% CI)	0.434

Abbreviations: Abbreviations: CONSORT=CONsolidated Standards for Reporting Trials; PR=Peer reviewer

© 2023 Speich B et al. *JAMA Network Open.*

eTable 5: The Difference in the Mean Proportion of Adequate Reporting of the 10 Selected Reporting Items, Stratified by Sample Size and Journal Impact Factor

	**CONSORT-PR trial**						**SPIRIT-PR trial**					
	**Intervention group: Reminder to peer reviewers**		**Control group**				**Intervention group: Reminder to peer reviewers**		**Control group**			
	**N**	**Proportion of adequately reported items (95% CI)**	**N**	**Proportion of adequately reported items (95% CI)**	**Mean difference (95% CI)**	**p-value**	**N**	**Proportion of adequately reported items (95% CI)**	**N**	**Proportion of adequately reported items (95% CI)**	**Mean difference (95% CI)**	**p-value**
**Planned sample size**												
≥100	82	73.5% (69.5-77.5%)	89	69.6% (64.6-74.5%)	4.0% (-2.4-10.4%)	0.22	68[a]	48.4% (43.7-53.1%)	71 [a]	47.9% (43.7-52.0%)	0.5% (-5.7-6.7%)	0.87
<100	40	60.8% (55.1-33.4%)	32	58.4% (51.8-65.1%)	2.3% (-6.2-10.9%)	0.59	19 [a]	37.9% (27.0-48.7%)	16 [a]	35.0% (25.5-44.5%)	2.9% (-11.3-17.1%)	0.68
**Journal impact factor[a]**												
≥10	34	83.8% (79.3-88.3%)	31	85.5% (81.0-90.0%)	1.7% (-4.6-7.9%)	0.60	NA	NA	NA	NA	NA	NA
<10	88	63.8% (59.9-67.6%)	90	60.1% (55.5-64.7%)	3.6% (-2.3-9.6%)	0.23	NA	NA	NA	NA	NA	NA

[a]Journal impact factor in 2020 according to the Journal Citation Reports

Abbreviations and interpretation: CONSORT=CONsolidated Standards for Reporting Trials; SPIRIT=Standard Protocol Items: Recommendations for Interventional Trials; PR=Peer reviewer; N=sample size; CI=confidence intervals.

© 2023 Speich B et al. *JAMA Network Open.*

eFigure: Histograms

Histograms visualizing the duration (days) from assigning an editor to the first decision was communicated for all randomized manuscripts from the CONORT-PR trial (a), only accepted manuscripts from the CONSORT-PR trial that were included in the analysis (b), all randomized manuscripts from the SPIRIT-PR trial (c), and only accepted manuscripts from the SPIRIT-PR trial that were included in the analysis.

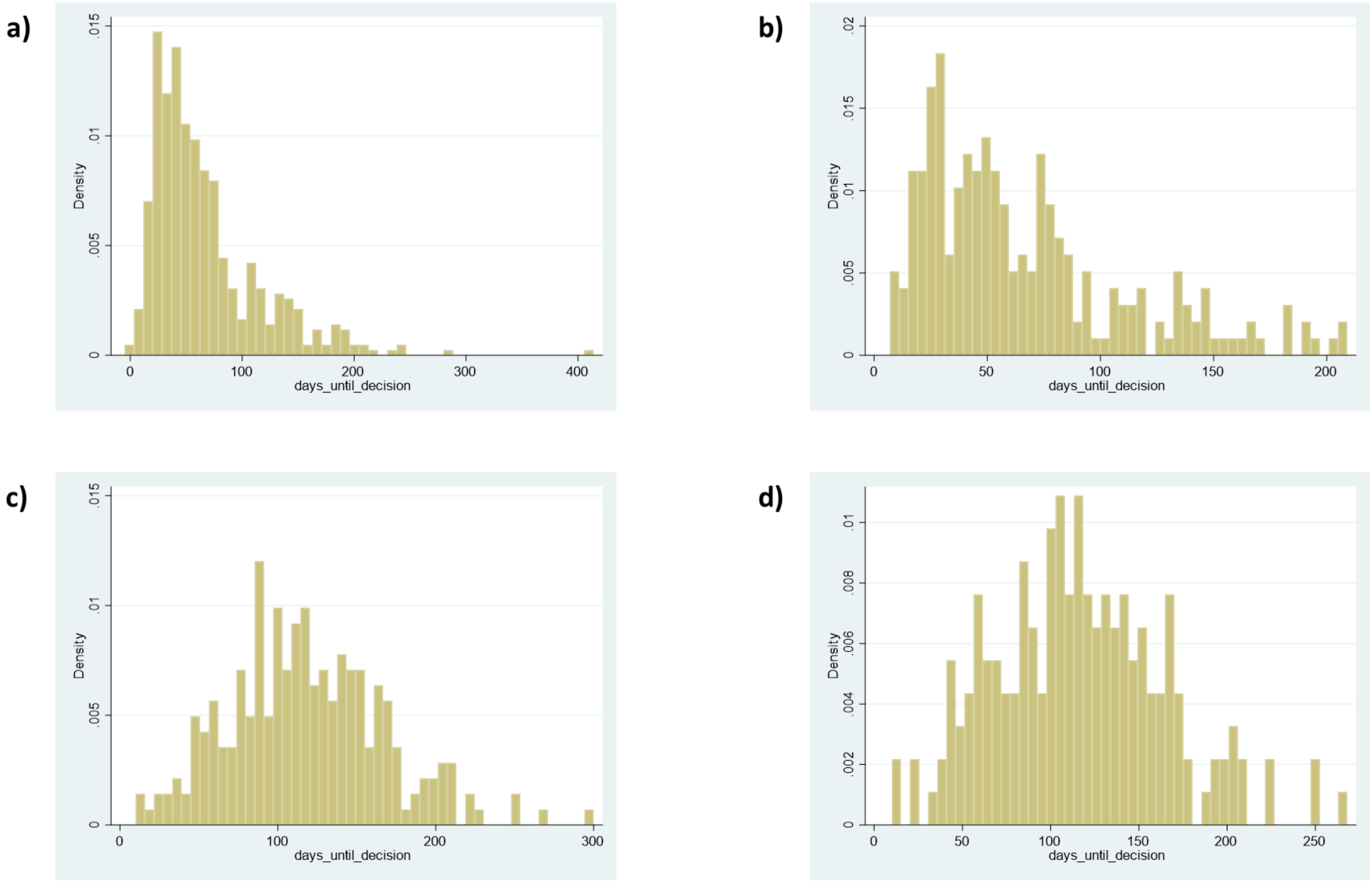

© 2023 Speich B et al. *JAMA Network Open.*

eReferences:

1. Speich B, Schroter S, Briel M, Moher D, Puebla I, Clark A, et al. Impact of a short version of the CONSORT checklist for peer reviewers to improve the reporting of randomised controlled trials published in biomedical journals: study protocol for a randomised controlled trial. BMJ Open. 2020;10(3):e035114.
2. Hopewell S, Boutron I, Altman DG, Barbour G, Moher D, Montori V, et al. Impact of a web-based tool (WebCONSORT) to improve the reporting of randomised trials: results of a randomised controlled trial. Bmc Med. 2016;14.
3. Moher D, Hopewell S, Schulz KF, Montori V, Gotzsche PC, Devereaux PJ, et al. CONSORT 2010 explanation and elaboration: updated guidelines for reporting parallel group randomised trials. BMJ. 2010;340:c869.
4. Kyte D, Duffy H, Fletcher B, Gheorghe A, Mercieca-Bebber R, King M, et al. Systematic evaluation of the patient-reported outcome (PRO) content of clinical trial protocols. PLoS One. 2014;9(10):e110229.
5. Speich B, Odutayo A, Peckham N, Ooms A, Stokes JR, Saccilotto R, et al. A longitudinal assessment of trial protocols approved by research ethics committees: The Adherance to SPIrit REcommendations in the UK (ASPIRE-UK) study. Trials. 2022;23(1):601.
6. Gryaznov D, von Niederhausern B, Speich B, Kasenda B, Ojeda-Ruiz E, Blumle A, et al. Reporting quality of clinical trial protocols: a repeated cross-sectional study about the Adherence to SPIrit Recommendations in Switzerland, CAnada and GErmany (ASPIRE-SCAGE). BMJ Open. 2022;12(5):e053417.
7. Chan AW, Tetzlaff JM, Gotzsche PC, Altman DG, Mann H, Berlin JA, et al. SPIRIT 2013 explanation and elaboration: guidance for protocols of clinical trials. BMJ. 2013;346:e7586.

© 2023 Speich B et al. *JAMA Network Open.*
