# Supplementary material for: Reminding Peer Reviewers of Reporting Guideline Items to Improve Completeness in Published Articles: Primary Results of 2 Randomized Trials
Source: JAMA Netw Open. 2023 Jun 9;6(6):e2317651. doi: 10.1001/jamanetworkopen.2023.17651 (PMC10257091; doi:10.1001/jamanetworkopen.2023.17651)
Supplement: Supplement 5. — Data Sharing Statement [file jamanetwopen-e2317651-s005.pdf]

# Data Sharing Statement

Speich. Reminding Peer Reviewers of Reporting Guideline Items to Improve Completeness in Published Articles. *JAMA Netw Open*. Published June 09, 2023.
doi:10.1001/jamanetworkopen.2023.17651

**Data**
**Data available:** No

**Additional Information**
**Explanation for why data not available:** The unit of randomization in our randomized controlled trial were not patients. Instead we randomized manuscripts that were submitted to biomedical journals. We made a limited dataset available, which will allow researchers to re-calculate the main outcomes. Due to confidentiality agreements with publishing journals we omitted variables from the dataset that could identify specific studies or journals (i.e. sample size, date of submission, medical field).
